# Mouse PRDM9 DNA-Binding Specificity Determines Sites of Histone H3 Lysine 4 Trimethylation for Initiation of Meiotic Recombination

**DOI:** 10.1371/journal.pbio.1001176

**Published:** 2011-10-18

**Authors:** Corinne Grey, Pauline Barthès, Gaëlle Chauveau-Le Friec, Francina Langa, Frédéric Baudat, Bernard de Massy

**Affiliations:** 1 Institut de Génétique Humaine, CNRS UPR 1142, Montpellier, France; 2 Centre d'Ingénierie Génétique Murine, Institut Pasteur, Paris, France; National Cancer Institute, United States of America

## Abstract

Meiotic recombination generates reciprocal exchanges between homologous chromosomes (also called crossovers, COs) that are essential for proper chromosome segregation during meiosis and are a major source of genome diversity by generating new allele combinations. COs have two striking properties: they occur at specific sites, called hotspots, and these sites evolve rapidly. In mammals, the *Prdm9* gene, which encodes a meiosis-specific histone H3 methyltransferase, has recently been identified as a determinant of CO hotspots. Here, using transgenic mice, we show that the sole modification of PRDM9 zinc fingers leads to changes in hotspot activity, histone H3 lysine 4 trimethylation (H3K4me3) levels, and chromosome-wide distribution of COs. We further demonstrate by an in vitro assay that the PRDM9 variant associated with hotspot activity binds specifically to DNA sequences located at the center of the three hotspots tested. Remarkably, we show that mutations in *cis* located at hotspot centers and associated with a decrease of hotspot activity affect PRDM9 binding. Taken together, these results provide the direct demonstration that *Prdm9* is a master regulator of hotspot localization through the DNA binding specificity of its zinc finger array and that binding of PRDM9 at hotspots promotes local H3K4me3 enrichment.

## Abstract 2

The nature of the PRDM9 zinc finger domain determines the location of hotspots for meiotic recombination in the genome and promotes local histone H3K4 trimethylation.

## Author Summary

Meiosis is the process of cell division that reduces the number of chromosome sets from two to one, so producing gametes for sexual reproduction. During meiosis in many organisms, there is reciprocal exchange of genetic material between homologous chromosomes by the formation of “crossovers,” which promote genetic diversity by creating new combinations of gene variants and play an important mechanical role in the segregation of chromosomes. Crossovers do not occur randomly throughout the genome, but in small regions called hotspots. Recent work showed that hotspots have specific structural features and that the protein PRDM9 is important in specifying their location. PRDM9 contains a so-called zinc finger domain that is predicted to bind specific DNA sequences, suggesting that hotspots might be sites where PRDM9 binds. By using transgenic mice expressing PRDM9 with modified zinc fingers, here we show directly that the nature of the zinc fingers in PRDM9 determines crossover hotspot localization. We show that PRDM9 binds DNA sequences at the center of hotspots. Furthermore, we identify DNA sequence polymorphisms that affect its binding and the extent of crossover activity. Overall, our work shows that PRDM9, through its zinc finger domain, is a master regulator of hotspot location in the mouse genome.

## Introduction

Meiotic recombination generates reciprocal exchanges between homologous chromosomes (also called crossovers, COs) that are essential for proper chromosome segregation during meiosis and are a major source of genome diversity by generating new allele combinations. COs are not distributed randomly along chromosomes, but are clustered within short intervals (1 to 2 kb long in mice and humans) called hotspots, which result from the preferred initiation of meiotic recombination at specific sites (reviewed in [1]–[3]). In mammals, several hotspots were identified with methods allowing direct measurements of recombination frequencies [4]–[7], and a human genome-wide map of hotspots, with estimated recombination frequencies, was obtained based on patterns of linkage disequilibrium [8],[9]. A major challenge has been to search for specific features of hotspots and to identify factors controlling their location. While no DNA sequence unambiguously associated with hotspot activity had been found before, the population diversity analysis uncovered a few short sequence motifs, which were overrepresented at CO hotspots [9]. A further refinement of the analysis revealed that one of them, the partially degenerated 13-mer CCNCCNTNNCCNC, was associated with 41% of 22,700 LD-based hotspots identified in the human genome. Within and around this motif, the most conserved bases showed a 3 bp periodicity, reminiscent of the 3 bp binding unit of C2H2 zinc fingers [10]. In addition, a chromatin analysis at two mouse hotspots revealed that hotspot activity was correlated with H3K4me3 enrichment at their center [11].

Interestingly, the *Prdm9* gene (also known as *Meisetz*) encodes for a protein with an array of C2H2 zinc fingers, catalyzes the trimethylation of the lysine 4 of histone H3 (H3K4me3), and is essential for progression through meiotic prophase in mice [12]. The zinc finger array of the human major isoform of PRDM9 was shown to recognize the 13-mer DNA motif associated with human meiotic recombination hotspots, suggesting that PRDM9 sequence-specific binding to DNA could play a role in specifying the sites of meiotic recombination [10],[13],[14]. This hypothesis was supported by the correlation between variations in the PRDM9 zinc finger array and hotspot usage in mice and humans [13],[15]–[17].

The correlations observed in mice were based on comparisons of hotspot activities in mice carrying different haplotypes over a several Mb region, overlapping the *Prdm9* gene. These regions were named *Dsbc1* (4.6 Mb) and *Rcr1* (6.3 Mb) in the two studies where they had been reported [18],[19]. Specifically, the presence of the *wm7* allele of *Dsbc1* (from *Mus musculus molossinus*) correlates with high recombination rate at two hotspots (*Psmb9* and *Hlx1*) and with local H3K4me3 enrichment at the center of these hotspots in spermatocytes [11],[18]. Mice with the *Dsbc1^wm7^* allele also show a different genome-wide distribution of COs in comparison to strains carrying the *Dsbc1^b^* allele (for instance, the C57BL/6 [hereafter B6] and C57BL/10 [B10] strains). Remarkably, the *Prdm9^b^* and *Prdm9^wm7^* alleles differ by their number of zinc fingers (12 and 11, respectively) and by 24 non-synonymous substitutions, which are all, but one, localized in the zinc finger array [13]. Whether the polymorphisms in the zinc finger array are responsible for these observed effects or whether other loci in the interval defining *Dsbc1* could contribute to the control of hotspot distribution remained to be determined.

Here, using transgenic mice, we establish that changing the identity of PRDM9 zinc fingers is sufficient to change hotspot activity, histone H3 lysine 4 trimethylation (H3K4me3) levels at the hotspots tested, and chromosome-wide distribution of COs. We further demonstrate using in vitro assays that PRDM9 variants bind to DNA sequences located at the center of the hotspots they activate. Taken together, these results demonstrate that *Prdm9* is a master regulator of hotspot localization in mice, through the DNA binding specificity of its zinc finger array.

## Results and Discussion

### CO Hotspots Are Specified by the PRDM9 Zinc Finger Array

To demonstrate that the hotspot features of *Dsbc1* are due to the identity of the PRDM9 zinc finger array and not to flanking genetic elements, we modified the *Prdm9^b^* allele of a B6 Chromosome 17 genomic fragment inserted in a bacterial artificial chromosome (BAC) by replacing its zinc finger array with that of the *Prdm9^wm7^* allele. This modified allele was named *Prdm9^wm7ZF^*. Transgenic mice were produced by micro-injection in fertilized one-cell B6 embryos of the BAC containing the *Prdm9^wm7ZF^* allele (hereafter Tg(wm7)) or the unmodified *Prdm9^b^* allele (hereafter Tg(b)) as a control (Table 1). *Prdm9* carried by the transgenes was expressed at a level slightly lower (Tg(wm7), strain #43) or similar (Tg(b), strain #75) to that of endogenous *Prdm9* (see Figure S1). We then asked whether the expression of *Prdm9^wm7ZF^* was sufficient to recapitulate the *Dsbc1^wm7^* phenotype concerning the recombination rate at the *Psmb9* hotspot, the enrichment of H3K4me3 at the *Psmb9* and *Hlx1* hotspots, and the distribution of COs along one whole chromosome.

**Table 1:** Mouse strains.

Name	*Prdm9* Genotype	Transgene	*Psmb9* Hotspot Genotype (SNPs at Hotspot Center)		Reference
B6	*Prdm9^b/b^*	—	*b/b*	(*TC/TC*)	
B10.A	*Prdm9^b/b^*	—	*a/a*	(*TC/TC*)	
R209	*Prdm9^wm7/wm7^*	—	*wm7-a/wm7-a*	(*TC/TC*)	[36]
RB2	*Prdm9^wm7/wm7^*	—	*b/b*	(*TC/TC*)	[18]
RJ2	*Prdm9^wm7/wm7^*	—	*b/b*	(*TC/TC*)	[18]
B6-Tg(b)	*Prdm9^b/b^*	*Prdm9^b^*	*b/b*	(*TC/TC*)	This work
B6-Tg(wm7)	*Prdm9^b/b^*	*Prdm9^wm7ZF^*	*b/b*	(*TC/TC*)	This work
B10.MOL(SGR)	*Prdm9^wm7/wm7^*	—	*wm7/wm7*	(*CT/CT*)	[40]

The relevant genotype of the mouse strains used in this study are indicated. At the *Prdm9* locus, strains carry the *b* allele (from *b* haplotype, present in strains such as B6) and/or the *wm7* allele (from *wm7* haplotype, from the *M. musculus molossinus* derived strain B10. MOL(SGR)). The *Prdm9* transgenes carry the zinc finger array either from the *b* or *wm7* alleles (see Materials and Methods). At the *Psmb9* hotspot, alleles are from *b*, *a* (from the B10.A strain), *wm7*, or the recombinant *wm7-a* haplotype. The relevant single nucleotide polymorphisms (SNPs) at the center of the hotspot are shown.

**Figure 1:**
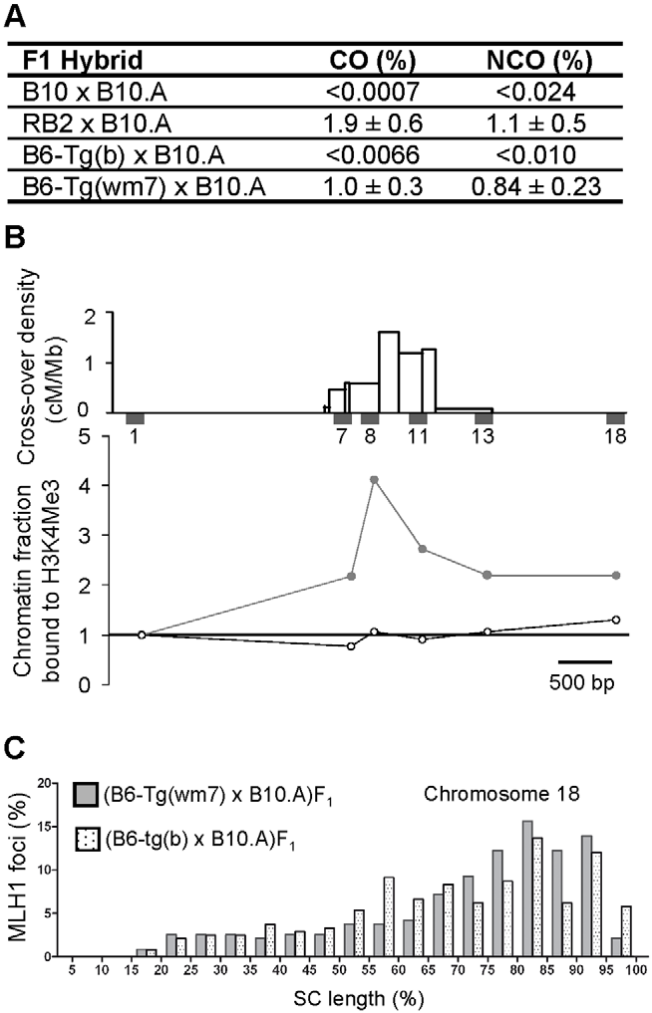
Influence of the PRDM9 zinc fingers on meiotic recombination and H3K4 trimethylation at recombination hotspots. (A) Recombination activity at the *Psmb9* hotspot is controlled by the PRDM9 zinc finger array. COs and NCOs at site “*38*” were measured by sperm typing [20]. The 95% confidence intervals for recombinant product frequencies are calculated as described in [20]. The difference in CO frequency between RB2×B10.A and B6-Tg(wm7)xB10.A is marginally significant (*p* = 0.03, two-sided heteroscedastic Student's *t* test). Values for B10×B10.A are from [20] and for RB2×B10.A from [18]. (B) H3K4me3 enrichment at the *Psmb9* hotspot is controlled by the PRDM9 zinc finger array. Top panel, distribution of COs and positions of STSs used for chromatin analysis along the *Psmb9* hotspot, from [39]. The chromatin fraction bound to H3K4me3, normalized to the sequence-tagged site (STS) Psmb9-1 (STS1, the 5′ most flanking STS), was determined for each STS, as described in [11]. Open circles, (B6-Tg(b)×B10.A)F_1_; grey circles, (B6-Tg(wm7)×B6)F_1_. The statistical analysis is shown in Table S2. (C) CO chromosome-wide distribution is controlled by the PRDM9 zinc finger array. The distribution of MLH1 foci along Chromosome 18 was determined as described in [18] in pachytene chromosome spreads from (B6-Tg(b)×B10.A)F_1_ (spotted columns) and (B6-Tg(wm7)×B10.A)F_1_ (grey columns) hybrids. Each column represents the percentage of MLH1 foci per 5% interval of SC length. According to the size of chromosome 18 (90.722.031 bp, NCBI m37), one interval corresponds to about 4.5 Mb. Results for the two hybrids are comparable to the CO distribution observed in (B10×B10.A)F_1_ and (RB2×B10.A)F_1_ hybrids, respectively, but are significantly different from each other (Figure S3 and Table S4).

First, the recombination rate at *Psmb9* was measured by sperm typing in (B6-Tg(wm7)×B10.A) and in (B6-Tg(b)×B10.A) F_1_ mice (Figure 1A and Table S1). In (B6-Tg(wm7)×B10.A) F_1_ mice, COs and non-crossovers (NCOs) frequencies were high at the *Psmb9* hotspot, like in hybrids with a *Dsbc1^wm7^* allele (such as the (RB2×B10.A)F_1_ hybrid, Figure 1A). The RB2 strain carries the *Dsbc1^wm7^* allele, together with the *b* haplotype at the *Psmb9* hotspot like the B6 and B10 strains (see Material and Methods and Table 1). Conversely, there was no detectable recombination at *Psmb9* in (B6-Tg(b)×B10.A)F_1_ mice, like in (B10×B10.A)F1 hybrids. Therefore, expression of the *Prdm9^wm7ZF^* allele is sufficient to activate the *Psmb9* recombination hotspot. We then determined the level of H3K4me3 at the *Psmb9* and *Hlx1* hotspots in spermatocytes from mice carrying Tg(b) or Tg(wm7). Spermatocytes from (B6-Tg(b)×B10.A) F_1_ mice did not display any local enrichment for H3K4me3, similarly to spermatocytes from the recombinationally inactive B6 strain (Figure 1B, Figure S2, Tables S2 and S3) [11]. Conversely, H3K4me3 was significantly enriched at the center of both hotspots in spermatocytes from (B6-Tg(wm7)×B6) F_1_ mice, similarly to the R209 strain, in which both hotspots are active [11]. We then compared the chromosome-wide distribution of COs, based on the mapping of MLH1 foci along Chromosome 18 in spermatocytes from mice carrying Tg(b) or Tg(wm7) (Figure 1C). These distributions were significantly different (Tables S4 and S6) as well as the one of B6-Tg(b)xB10.A compared to RB2×B10.A (expressing the *Prdm9^wm7^* allele) and the one of B6-Tg(wm7)xB10.A compared to B10×B10.A (expressing only the *Prdm9^b^* allele) (Figure S3, Tables S4 and S5). In contrast, the distributions of MLH1 foci of the Tg(b) and Tg(wm7) transgenic strains were not different from that of strains expressing *Prdm9^b^* (B10×B10.A) and *Prdm9^wm7^* (RB2×B10.A), respectively (Figure S3, Table S4). Therefore, the expression of *Prdm9^wm7ZF^* is sufficient to promote a *wm7*-specific chromosome-wide distribution of COs.

### PRDM9 Binds In Vitro to Hotspot Sequences

**Figure 2:**
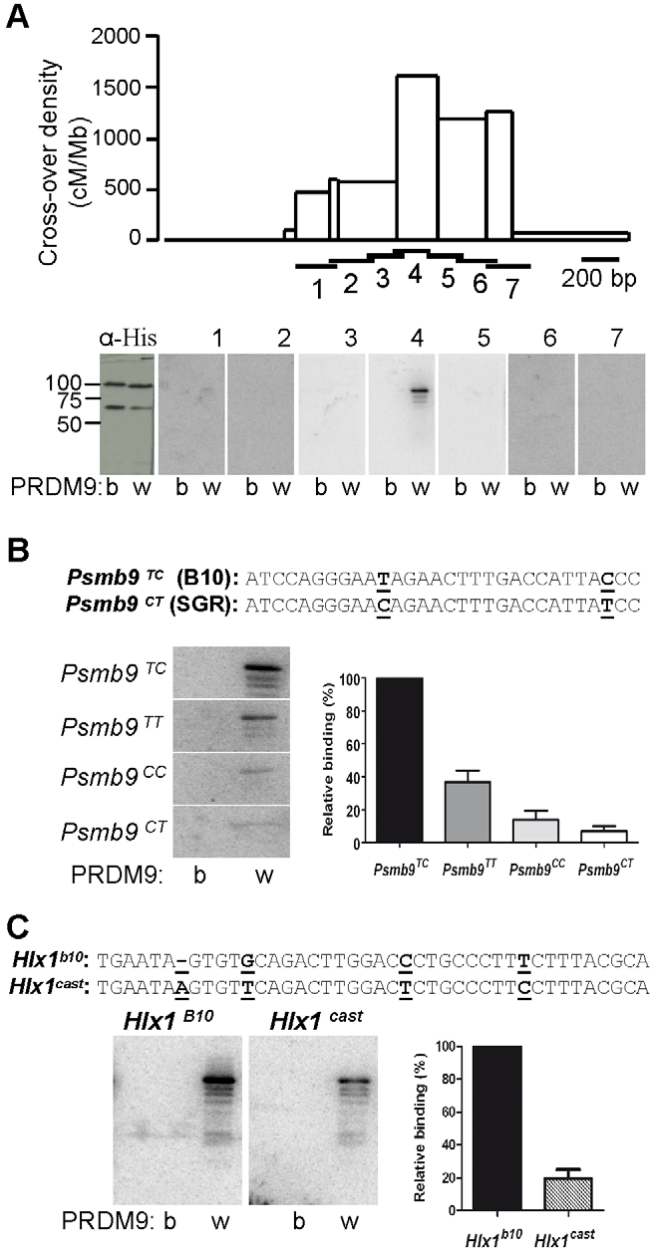
PRDM9 binds in vitro to meiotic recombination hotspots. (A) Detection by southwestern blotting of PRDM9 binding at the *Psmb9* recombination hotspot. Upper panel, CO distribution along the *Psmb9* hotspot [39]. Horizontal bars show the positions of the DNA probes (numbered from 1 to 7) used for southwestern experiments. Lower panel, PRDM9 (b, His-PRDM9^b^; w, His-PRDM9^wm7^) was probed with anti-His antibody and the radio-labeled double-stranded DNA probes 1–7 (about 200 bp). The molecular weights of His-PRDM9^b^ and His-PRDM9^wm7^ are 101 kDa and 98 kDa, respectively. The bands with lower molecular weights correspond to PRDM9 degradation products. (B) Effect of SNPs at the center of the *Psmb9* hotspot on the in vitro binding of PRDM9. The sequence of the likely PRDM9^wm7^ binding sequence [13] is shown, and the SNPs between the B10 and B10.MOL-SGR strains are underlined (see FigS5 for in silico prediction). PRDM9^b^ and PRDM9^wm7^ were probed with radio-labeled double-stranded oligonucleotides that carried the four possible SNP combinations (60 bp, Table S14). The amount of signal due to binding of each probe to PRDM9^wm7^ is shown (with standard error), relative to *Psmb9^TC^*. The decrease of binding to the double mutant probe *Psmb9* ^CT^ (0.07% binding relative to *Psmb9* ^TC^) is consistent with a cumulative effect of each single mutant (0.36% and 0.14% binding relative to *Psmb9* ^TC^) suggesting their effects are independent. (C) Analysis by southwestern blotting of PRDM9 binding to the putative PRDM9^wm7^ binding motif at the center of the *Hlx1* hotspot [13]. The likely PRDM9^wm7^ binding sequences in the B10 and CAST strains are shown, with SNPs underlined (see Figure S5 for in silico prediction). PRDM9^b^ and PRDM9^wm7^ were probed with radio-labeled double-stranded oligonucleotides that carried B10 or CAST allele (41 bp, Table S14). Signal intensities of the binding of the *Hlx1^B10^* and *Hlx1^cast^* probes (relative to *Hlx^B10^*) to PRDM9^wm7^ are shown.

**Figure 3:**
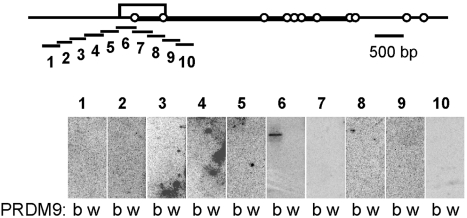
PRDM9 binds to the *G7c* recombination hotspot. Top, map of the genomic region of the *G7c* hotspot, located in the seventh intron of the D6S56E-3 gene on Chromosome 17. The open box represents the 800 bp interval with the highest density of exchanges, as mapped in [22]. The open circles indicate the positions of the SNPs that are polymorphic in the hybrids used for measuring recombination (*G7c^b/a^*, see Table S7). The interval drawn as a thick line is the interval amplified by allele-specific PCR for measuring the recombination frequencies shown on Table S7. The position of the 10 probes used for southwestern blotting is shown underneath. Bottom, PRDM9 (b, His-PRDM9^b^; w, His-PRDM9^wm7^) was probed with the radio-labeled double-stranded DNA probes 1–10 (about 250 bp).

In order to show that these effects are due to a direct interaction between PRDM9 and hotspot DNA sequences, we tested in vitro the binding of different PRDM9 variants to hotspot regions. We first examined the binding of recombinant His-tagged PRDM9^wm7^ and PRDM9^b^ to a series of overlapping DNA fragments that covered 1.3 kb across the *Psmb9* hotspot. Strikingly, PRDM9^wm7^, but not PRDM9^b^, bound to a single DNA fragment located at the center of this hotspot (Figure 2A). This 200 bp DNA fragment contains a 31 bp sequence with a partial match (*p* = 2.43×10^−3^, Figure S4, Text S1) to the predicted PRDM9^wm7^ binding site. PRDM9^wm7^ could also bind to a 61 bp double-stranded oligonucleotide that contained this sequence (Figure 2B, probe *Psmb9^TC^*). Furthermore, in the B10.MOL-SGR strain, in which this sequence differs by two single nucleotide polymorphisms (SNPs) from the one of the B10 strain, recombination initiation rate at *Psmb9* is at least 10 times lower than in B10 mice [20]. In vitro binding assays showed that these two SNPs affected independently the binding of PRDM9^wm7^ to the double-stranded oligonucleotide (Figure 2B). Thus, both variation in the zinc finger array of PRDM9 and polymorphisms in the target sequence are involved in the control *in trans* and *in cis* of the recombination rate at the *Psmb9* hotspot. Additionally, we examined the binding of PRDM9 to the *Hlx1* hotspot on Chromosome 1, the activity of which depends on the presence of the *wm7* or *cast* haplotype at *Dsbc1* (both haplotypes have the *Prdm9^wm7^* allele [18],[19]) and in which the level of H3K4me3 was increased in the presence of *Prdm9^wm7ZF^* (Figure S2). At *Hlx1*, PRDM9^wm7^, but not PRDM9^b^, could bind to a motif localized at the center of the hotspot (Figure 2C). Interestingly, the B10 and CAST/EiJ (*M. m. castaneus*) strains are polymorphic for that motif, and the distribution of COs across this hotspot in a hybrid carrying one chromosome from each strain indicates that the initiation rate is approximately double on the B10 chromosome than on the CAST chromosome [7],[11]. In line with this variation, PRDM9^wm7^ had a higher affinity for the B10 sequence than for the CAST one (Figure 2C). The sensitivity to small changes in the PRDM9 target sequence might explain why the recombination rate at hotspots is exquisitely sensitive to either polymorphisms in *cis* or to subtle changes within the zinc finger array of PRDM9 [15],[21]. We also examined the reciprocal situation where a hotspot (the *G7c* hotspot on Chromosome 17) is active in the presence of the *b* allele of *Prdm9* [22]. We determined by sperm typing that the recombination rate at the *G7c* hotspot was at least 30-fold higher in *Prdm9^b/b^* than in *Prdm9^wm7/wm7^* mice (Table S7). By examining in vitro the binding of PRDM9 to 10 overlapping DNA fragments covering 2.2 kb along the *G7c* hotspot, we found that PRDM9^b^ bound to a single fragment mapping to the interval with the highest exchange density, whereas no binding of PRDM9^wm7^ could be detected (Figure 3). Taken together, these results demonstrate that PRDM9 recognizes specific DNA sequences that are localized at the center of the three recombination hotspots tested. Surprisingly, the in vitro binding specificity we detected was not predicted by the C2H2 zinc finger prediction program [23]. In particular, the *Psmb9* and *G7c* DNA probes showing binding to PRDM9 did not contain any significant match (with a *p* value<10^−3^) to the predicted PRDM9 motif, whereas significant matches were predicted in regions where no in vitro binding could be detected (Figure S4, Tables S8 and S9).

### Kinetics of *Prdm9* Expression and H3K4me3 Enrichment

**Figure 4:**
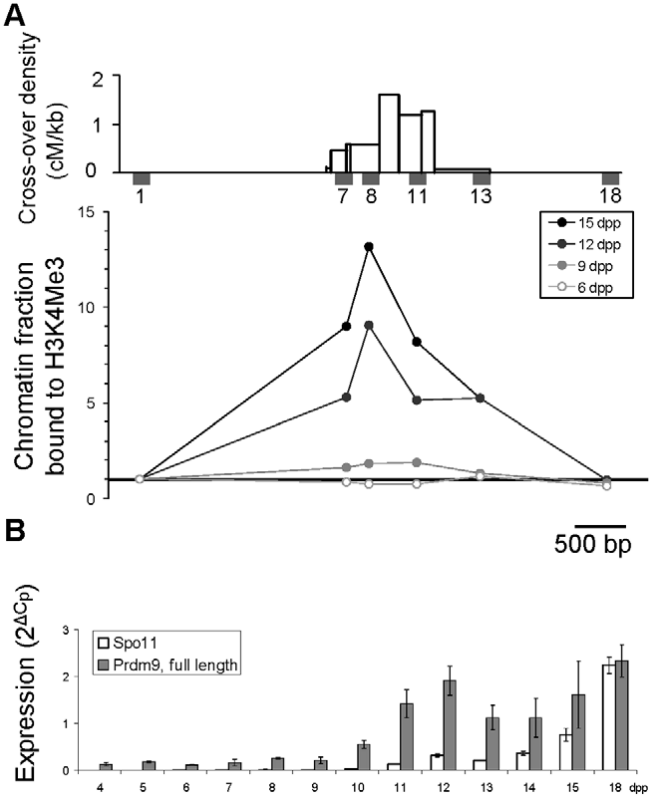
Kinetics of H3K4 trimethylation and *Prdm9* expression in testes of prepuberal mice. (A) Top panel, distribution of COs and positions of STSs used for chromatin analysis along the *Psmb9* hotspot, from [39]. The fraction of chromatin bound to H3K4me3, normalized to STS1 (the 5′ most flanking STS), was determined along the *Psmb9* hotspot in whole testes from prepuberal R209 mice, as described previously [11]. Data in 9 dpp mice are from [11]. (B) Steady-state levels of *Spo11* (white) and *Prdm9* (full length, gray) transcripts were determined in whole testes from 4 to 18 dpp R209 mice. Given that the first wave of entry into meiosis is relatively synchronous, the decrease in *Prdm9* transcript levels at 13 and 14 dpp may indicate a transient expression of *Prdm9* at the beginning of meiotic prophase (10–12 dpp). The significant increase detected later at 18 dpp parallels the second wave of entry into meiosis.

If PRDM9 is responsible for the H3K4me3 mark that defines initiation sites of meiotic recombination, H3K4me3 enrichment should appear concomitantly with the onset of *Prdm9* expression at the time or before meiotic DNA double-strand break (DSB) formation [12]. Therefore, we examined the kinetics of *Prdm9* expression and of H3K4me3 at the *Psmb9* and *Hlx1* hotspots during the first wave of entry into meiosis in testes of prepuberal *Prdm9^wm7/wm7^* mice. During this wave, B-type spermatogonia enter meiosis at day 8–9 post-partum (8–9 dpp) and reach the leptotene stage of meiotic prophase, when DSBs are generated, at 9–10 dpp [24]. Spermatocytes then progress through meiotic prophase to reach metaphase I at around 20 dpp [25]. At 9 dpp, a modest but significant H3K4me3 enrichment was observed that increased at 12 and 15 dpp (Figure 4A, Figure S5A and Tables S10 and S11). No H3K4me3 enrichment was detected at 6 dpp, suggesting that this histone post-translational modification is not apposed to recombination hotspots before entry into meiosis. We then examined by real-time RT-PCR the kinetics of expression of three previously described *Prdm9* splicing variants [12],[26] during the first wave of meiosis. Full-length *Prdm9*, which is the most abundant isoform, and the S1 variant were detected and expressed with similar kinetics, whereas the S2 variant was undetectable (Figure S5B). Full-length *Prdm9* was expressed at a low level at all time points, but increased significantly from 10 dpp (*p*<0.05 with every previous time point, two-sided Student *t* test) (Figure 4B). Altogether, these findings are consistent with the hypothesis that PRDM9 is responsible for apposing H3K4me3 to recombination hotspots at or before the time of meiotic DSB formation.

### From PRDM9 DNA Binding to DSB Formation

**Figure 5:**
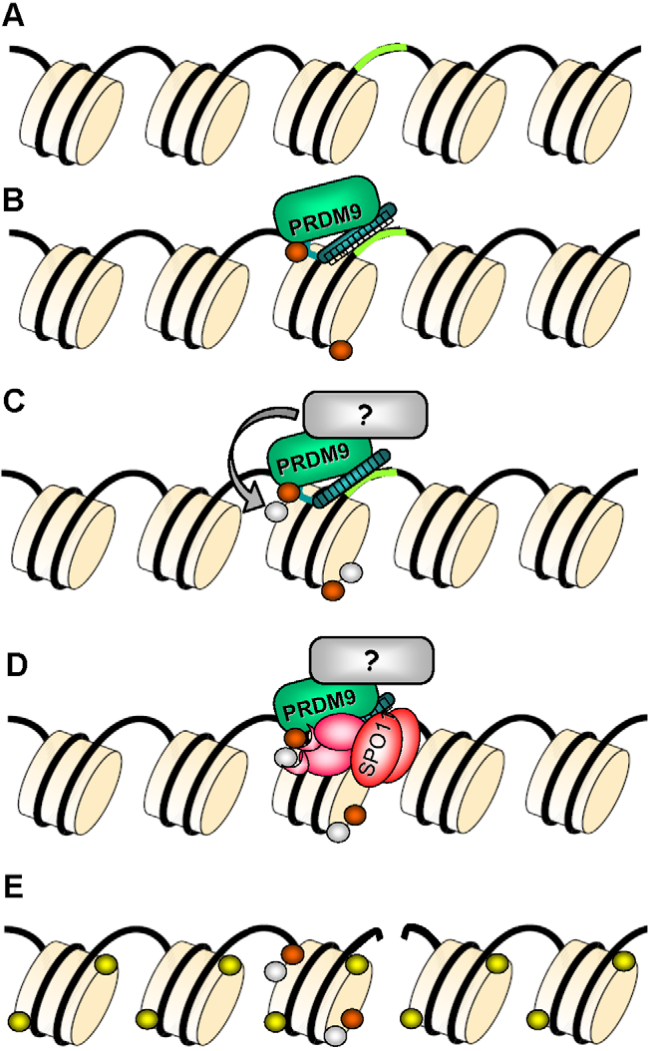
Model of hotspot specification by PRDM9. (A) The DNA and several nucleosomes are represented. A DNA sequence motif recognized by PRDM9 is represented in green. (B) PRDM9 binds to its target DNA motif through the zinc finger array and catalyzes H3K4me3 (orange). (C) A protein partner of PRDM9 may catalyze another post-translational histone modification (grey), allowing for the formation of a hotspot-specific signature. (D) PRDM9, a partner, or other component of the chromatin may recruit the recombination initiation complex containing SPO11 or may create a favorable chromatin environment allowing access of SPO11 to the DNA. (E) A DSB is formed by SPO11 and triggers the phosphorylation of histone H2Ax (yellow) in the surrounding nucleosomes. The DSB is then repaired by homologous recombination and lead to a CO or to gene conversion without CO.

Our results provide the first direct demonstration that the identity of the PRDM9 zinc finger array determines hotspot localization in mice through binding of PRDM9 to DNA sequences at hotspots and H3K4me3 enrichment at such regions. It is remarkable that, at all hotspots tested, the binding of PRDM9 occurs at or very near their center, suggesting a direct or highly localized interaction between PRDM9 activity and DSB formation. Our in vitro analysis also demonstrates the limitation of in silico prediction of PRDM9 DNA binding specificity, when applied to search for binding sites at individual hotspots. The complexity of the interaction between the PRDM9 zinc fingers and the DNA is obviously greater than the one analyzed for proteins containing smaller numbers of zinc fingers and used in the prediction algorithms. Several human hotspots which activity has been shown to depend on *PRDM9* do not contain a match to the predicted motif [15],[27]. This could be due to the limited power of motif prediction and to additional factors that influence PRDM9 binding and/or its accessibility to its binding sites. The enrichment for H3K4me3 at active recombination hotspots, which is unambiguously dependent on PRDM9, is also highly localized and catalyzed very likely by PRDM9 itself. PRDM9 binding may also lead to the recruitment of additional factors and other chromatin remodelers. In fact, additional histone post-translational modifications were detected at the *Psmb9* hotspot [11] and H3K4me3 is expected not to be sufficient for promoting hotspot activity as it is known to be associated with genomic functional elements that generally are not recombination hotspots (such as transcriptional promoters) [28],[29]. One should also point out the formal possibility that H3K4Me3 enrichment may not be required for hotspot activity. Overall, how these hotspot features allow the recruitment of the proteins involved in meiotic DSB formation remains to be understood (Figure 5). An additional implication for the close vicinity of PRDM9 binding to the hotspot center is that the PRDM9 binding site has a high probability to be included in gene conversion tracts during meiotic recombination. This feature is key to account for the drive against the motif observed in humans [14].

A growing set of data suggests that meiotic recombination occurs mainly at *Prdm9*-dependent hotspots in mammals [13],[15],[18],[19]. This view is further supported by a recent genome-wide survey of mouse recombination hotspots, which revealed that 87% of them were overlapping with testis-specific H3K4me3 marks [30]. Whether alternative pathways for the specification of a subset of initiation sites do exist remains to be determined. In addition, whether PRDM9 binds to genomic sites not associated to recombination can be envisioned. Indeed, one unexplained property of PRDM9 is its role in hybrid sterility, where a specific combination of *Prdm9* alleles differing in their zinc finger array leads to male-specific sterility, potentially as a result of a change in gene expression [26].

The *Prdm9* gene is well conserved among metazoans, however the domain encoding the zinc finger array experienced an accelerated evolution in several lineages, including rodents and primates [31],[32]. This accelerated evolution is restricted to codons responsible for the DNA-binding specificity of PRDM9 zinc fingers, which appear to have been subjected to positive selection [31],[32]. Surprisingly PRDM9 appears to have been lost from some lineages in animals [33], suggesting that alternative pathways may be used for specifying hotspots, such as the one described in the yeasts *Saccharomyces cerevisiae* and *Schizosaccharomyces pombe* where components of the transcription machinery are known to be involved in meiotic DSB formation [34],[35].

## Materials and Methods

### Mouse Strains

The mouse strains used in this study are C57BL/6NCrl (B6), C57BL/10JCrl (B10), B10.A-*H2^a^ H2-T18^a^*/SgSnJ (B10.A), B10.A(R209) (R209) [36], RB2, and RJ2. The RB2 strain results from backcrossing (B10×R209) F_1_ with B10 and carries the *wm7* haplotype on a *Prdm9*-containing interval of chromosome 17 [18]. The RJ2 strain is derived from RB2 and carries also the *Prdm9^wm7^* allele, as described in [18]. The mouse strains are shown on Table 1 with their genotypes at *Prdm9* and *Psmb9* hotspot. All experiments were carried out according to CNRS guidelines.

### Generation of Transgenic Mice

The bacterial artificial chromosome (BAC) RP23-159N6 containing an insert derived from C57BL/6J (Coordinates 15,651,974–15,848,091 on Chromosome 17, NCBI m37 mouse genome assembly) was obtained from the BACPAC Resource Center at the Children's Hospital Oakland Research Institute (Oakland, California, USA). The part of exon 12 encoding the PRDM9 zinc finger array was modified by BAC recombineering [37], using the primers MsGALKF and MsGALKR for the first step (Table S12). *GalK* was then replaced by the fragment encoding the *wm7* zinc finger array, which was generated by PCR amplification of B10.A(R209) genomic DNA with primers Pr1500U20 and Pr2848L23 (Table S12), resulting in the BAC RP23-159N6 (*Prdm9^wm7ZF^*). The last *Prdm9* exon, which encodes the zinc finger array, has been fully sequenced in both BACs.

Transgenic mice were generated by microinjection of 0.5–1 ng/microliter of circular BAC RP23-159N6(*Prdm9^wm7ZF^*) [Tg(wm7)] or RP23-159N6 [Tg(b)] into fertilized one-cell C57BL/6J embryos. Injected eggs were implanted in pseudopregnant (C57BL/6J×CBA) F1 foster mothers. Transgenic mice were identified by PCR analysis of mouse tail DNA using the primer pairs p3.6_1U and p3.6_1L, and p3.62U and p3.62L. Six pups integrated Tg(wm7) and seven Tg(b). Four mice with Tg(wm7) and seven with Tg(b) showed germ-line transmission. For Tg(wm7), one strain (#43, which contains two or three copies of the BAC, as determined by Southern blot) was used for all experiments, and similar results were obtained with another strain for CO measurement at *Psmb9* and H3K4me3 enrichment (not shown). For Tg(b), the distribution of MLH1 foci on Chromosome 18 was analyzed in strain #95, the recombination rate at *Psmb9* was determined in strains #55 and #95, and H3K4me3 enrichment was measured in strain #75, which contains four or five copies of the BAC, as determined by Southern blot. All transgenic mice used in this study were hemizygous for the transgene.

### Southwestern Blotting Assays and Cloning of His-Tagged Mouse PRDM9

Southwestern blotting assays were performed as described previously [13], using full-length His-tagged mouse PRDM9^wm7^ and PRDM9^b^. The *Prdm9^wm7^* and *Prdm9^b^* coding sequences were cloned as follows: cDNA prepared from C57BL/10Crl and R209 testis RNA was amplified with the primers 1S91U24 and Pr2848L23 (Table S13) using Phusion DNA polymerase (Finnzymes), as recommended by the supplier. Each PCR product was gel-purified and a second round of amplification was performed with 2 ng of purified product with the primers mPrdm9gwU and mPrdm9gwL. The products were gel-purified and integrated into the plasmid pDONR201 with BP clonase (Invitrogen). Then, the inserts containing the coding regions of *Prdm9^wm7^* and *Prdm9^b^* were transferred using LR clonase (Invitrogen) to the pET15bGtw expression vector, resulting in plasmids encoding N-terminally His-tagged PRDM9^wm7^ and PRDM9^b^ under the control of the T7 promoter. The insert sequences were then verified. For subsequent expression the plasmids were transformed into the BL21(DE3) *E. coli* strain.

The probes covering the *Psmb9* and *G7c* hotspots were generated by PCR amplification with *Xba*I site-tailed primers (Table S14). Amplification products were phenol/chloroform purified followed by ethanol precipitation, *Xba*I-digested, and agarose-gel purified. The probes containing a motif at the center of the *Psmb9* and *Hlx1* hotspots were made by annealing complementary oligonucleotides leaving a 3 or 4 bp 5′-overhang at each end (Table S14). DNA fragments were labeled by end-filling with alpha-^32^P dCTP as described previously [13].

### CO and NCO Measurements

At *Psmb9*, COs and NCOs at site *38* were measured in sperm DNA as described [20]. At *G7c*, semi-nested PCR was performed to detect the exchanges occurring in an interval overlapping with the genetically identified hotspot center [22]. PCR amplification was performed as for *Psmb9*, with the primers and annealing temperatures listed in Table S15.

The bias in CO distribution along the *Hlx1* hotspot in the (B10.A(R209)×CAST/Eij)F_1_ hybrid [11], which is homozygous for *Prdm9^wm7^*, results in a 68% segregation bias among the CO products that favors the CAST allele at the center of the hotspot. This segregation distortion indicates that the initiation rate on the B10 chromosome is approximately twice the one on the CAST chromosome in that hybrid.

### Chromosome-Wide CO Distribution

Chromosome spreads, fluorescent in situ hybridization (FISH), immunofluorescence (IF) assays, image acquisition, and statistical tests were performed as described [18]. Chromosome 18 was identified with a labeled BAC probe (RP23-101G16), and the following antibodies were used for the immunofluorescence assays: guinea pig anti-SYCP3 serum at 1∶500 dilution and mouse monoclonal anti-MLH1 (Pharmigen) at 1∶50 dilution.

### Chromatin Immunoprecipitation

Spermatocytes from testes of 3–4 adult mice were enriched by centrifugal elutriation as described [11]. Native chromatin was prepared from elutriated cells or from whole testis cells of prepuberal mice, immunoprecipitated with an antibody directed against H3K4me3 (rabbit polyclonal ab8580, Abcam), and immunoprecipitated DNA was quantified using real-time PCR as described [11]. As a control for the quality of the samples and of the immunoprecipitations, the level of H3K4me3 was measured at the *Actin*, *Sycp1*, and *Nestin* promoters. The sequences of the primers and PCR conditions for the studied STSs (Psmb9-1, -7, -8, -11, -13, and -18; Hlx1-1.2, -5, -6, -2.2, -3, and -4; *Actin*, *Nestin*, and *Sycp1* promoters) were described previously [11]. The Mann-Whitney test was used to determine the statistical significance of differences between strains for the data concerning the STSs 7, 8, 11, and 13 (*Psmb9*) or STSs 5, 6, and 2.2 (*Hlx1*) (Tables S2 and S3) or between time points (Tables S10 and S11).

### Expression Analyses

For determining the kinetics of expression in testes from prepuberal mice, total RNA from one testis from 4 to 18 dpp R209 mice was extracted with the GenElute Mammalian Total RNA Miniprep Kit (Sigma). Five hundred ng of RNA were reverse-transcribed with SuperscriptIII Reverse Transcriptase (Invitrogen) and random 10-mer primers. Two µl of cDNA at the appropriate dilution (see Table S16) was used for real-time PCR in a 10 µl reaction containing 1× LC480 SYBR Green mix (Roche) and 0.5 µM of the primers listed in Table S16, with PCR conditions as described [11]. The relative amount of each transcript of interest was determined with the 2^ΔCp^ method, using housekeeping genes (*Actin*, *Gapdh*, and *Hprt*) as a reference [38]. For determining the level of *Prdm9* RNA in transgenic mice, total RNA was extracted from elutriated cells from adult testes. The amount of *Prdm9* transcript was determined by using the same set of housekeeping genes, plus *Spo11*, as references (Figure S1A). The relative amount of RNA was quantified by using serial dilutions of RNA from a reference sample (B10 testis elutriated cells). To evaluate the relative amounts of endogenous *Prdm9^b^* RNA and *Prdm9^wm7ZF^* in B6-Tg(wm7) mice, a 1.3 kb interval encompassing the zinc finger array-coding domain was amplified from the cDNA (primers Pr1500U20 and Pr2848L23, Table S12) and run on an agarose gel in conditions that discriminate both alleles (amplicon of 1,371 bp for *Prdm9^b^*, 1,287 bp for *Prdm9^wm7^*). The relative amounts of *Prdm9^b^* and *Prdm9^wm7ZF^* RNAs were compared to a sample resulting from amplifying genomic DNA from a *Prdm9^b/wm7^* mouse, which contains the same amount of both alleles (Figure S1B).

## Supporting Information

Figure S1 Expression of transgenic *Prdm9* copies. (A) The level of *Prdm9* transcript in total RNA from elutriated testis cells was measured by RT-qPCR, using *Gapdh*, *Hprt*, *Actin*, and *Spo11* as references. The ratio was normalized to 1 for the average of the four samples from strains without a transgene (B10 and RJ2). The two B10 and RJ2 samples are independent preparations from different mice of the same genotype. (B) A 1,371 bp (allele *b*) or 1,287 bp (allele *wm7*) fragment of *Prdm9* cDNA was amplified from several cDNA samples and run on an agarose gel. Controls without reverse-transcriptase (RT) show no amplification. The *Prdm9^b/wm7^* genomic DNA sample provides a reference for equimolar concentration of both alleles, showing the more efficient amplification of the smaller *wm7* allele. The amounts of product of both alleles appear fairly similar in the sample from the transgenic B6-Tg(wm7) strain #43, indicating that there is slightly more RNA from the endogenous *Prdm9^b^* locus than from the *Prdm9^wm7ZF^* transgene. Size markers of 1,371 and 1,264 bp are indicated by arrows. n.a., not applicable.
(TIF)

Figure S2 H3K4me3 enrichment at the *Hlx1* hotspot is controlled by the PRDM9 zinc finger array. Top panel, distribution of COs and positions of STSs along the *Hlx1* hotspot [2]. The chromatin fraction bound to H3K4me3, normalized to *Psmb9* STS1 (the 5′ most flanking STS at the *Psmb9* hotspot), was determined in elutriated spermatocytes for each STS, as described [2]. Open circles, (B6-Tg(b)×B10.A)F_1_; gray circles, (B6-Tg(wm7)×B6)F_1_.
(TIF)

Figure S3 Distribution of MLH1 foci along chromosome 18. The distribution of MLH1 foci along chromosome 18 was determined in pachytene chromosome spreads as described [3]. White, (B10×B10.A)F_1_, data from [3]; black, (RB2×B10.A)F_1_, data from [3]; spotted, (B6-Tg(b)×B10.A)F_1_; gray, (B6-Tg(wm7)×B10.A)F_1_.
(TIF)

Figure S4 Prediction of DNA sequence motifs recognized preferentially by PRDM9^b^ and PRDM9^wm7^. The predictions of the DNA binding sequences of PRDM9^b^ and PRDM9^wm7^ were generated using the program developed by Persikov et al. (http://zf.princeton.edu/) [1]. The logos for the sequences predicted to bind PRDM9^b^ and PRDM9^wm7^ are shown. Under the PRDM9^b^ logo is the best matching sequence in the interval covered by the *G7c* probe 6, which binds PRDM9 in vitro (Figure 3). The sequences bound in vitro to PRDM9^wm7^ at the center of hotspots *Psmb9* and *Hlx1* are aligned under the PRDM9^wm7^ logo. The matching residues are in bold, and the polymorphisms affecting PRDM9 binding in vitro and recombination initiation in vivo are underlined (see Figure 2B and C). The *p* values given by the FIMO program are shown.
(TIF)

Figure S5 Kinetics of H3K4 trimethylation at *Hlx1* and *Prdm9* expression in testes of prepuberal mice. (A) Top panel, distribution of COs and positions of STSs along the *Hlx1* hotspot [2]. The chromatin fraction bound to H3K4me3, normalized to STS1 (the 5′ most flanking STS), was determined along the *Hlx1* hotspot in whole testes from prepuberal R209 mice, as described [2]. (B) Top panel, steady-state levels of *Spo11* (white) and *Prdm9* (all splicing variants, black; full length, grey; S1 splicing variant, white) expression were determined in whole testes from 4 to 18 dpp mice. The relative changes in expression of the S1 variant are also shown in the lower panel in which a scale with a lower order of magnitude was used.
(TIF)

Table S1 Measurement of CO and NCO at the *Psmb9* hotspot in sperm from (B6-Tg×B10.A) F1 mice. CO A-B and CO B-A indicate exchange products in B10.A to B6 and B6 to B10.A orientation, respectively. NCO A→B indicates non-crossover events having taken place on the B10.A chromosome. Reciprocally, NCO B→A indicates the non-crossover events that took place on the B6 chromosome (see [4]).
(DOC)

Table S2 Statistical analysis of H3K4me3 enrichment in elutriated spermatocytes from transgenic mice. Inter-genotype statistical analysis of H3K4me3 enrichment (values shown on Table S3) in elutriated spermatocytes on *Psmb9* and *Hlx1* hotspots. Stars indicate significant statistical difference (*p*<0.05) between the genotypes. Data for B6 and R209 were imported from [2]. The level of H3K4 enrichment at *Psmb9* and *Hlx1* hotspots in purified spermatocytes was compared between transgenic mice and non-transgenic mice of various *Prdm9* genotypes. ^a^ The difference observed at *Hlx1* reflects lower H3K4me3 enrichments in B6-Tg (b)×B10.A as compared to B6 (see values in Table S3).
(DOC)

Table S3 H3K4me3 enrichment in elutriated spermatocytes from transgenic mice at hotspots *Psmb9* and *Hlx1*. The values in Table S3 are the bound fraction for each STS, normalized to the bound fraction for STS Psmb9-1, as described in [2]. B6 and R209 data are from [2].
(DOC)

Table S4 Statistical analysis of the variation between mouse strains in the MLH1 focus distribution on chromosome 18. The distribution of MLH1 foci along chromosome 18 synaptonemal complex (SC) was compared between spermatocytes from mice with different genotypes, using a nonparametric Kolmogorov-Smirnov test and a chi-square test. Stars indicate significant statistical difference (*p*<0.05) between genotypes. We showed previously for (B10×B10.A) and (RB2×B10.A) F1 hybrids that the distribution of MLH1 foci did not vary significantly between individuals of the same genotype (see Table S7 in [3]). Data for B10×B10.A and RB2×B10.A were imported from [3].
(DOC)

Table S5 SC lengths, average, and total MLH1 focus number on chromosome 18. Data for B10×B10.A and RB2×B10.A were imported from [3].
(DOC)

Table S6 Distributions of MLH1 foci on chromosome 18 in transgenic mice. The number of MLH1 foci per 5% interval of chromosome 18 synaptonemal complex length is shown for B6-Tg(b)xB10.A and B6-Tg(wm7)xB10.A mice.
(DOC)

Table S7 Exchange frequency at the *G7c* hotspot depends on the *Prdm9* allele.
(DOC)

Table S8 Predicted PRDM9^b^ and PRDM9^wm7^ binding sequences with a *p* value lower than 10^−3^ at *G7c*, *Psmb9*, and *Hlx1* hotspots. The scoring matrices resulting from the predictions (see Text S1 and Figure S4) were used for searching the intervals that have been probed by South-Western blot (*G7c* and *Psmb9*), or a 2 kb interval centered on the hotspot center (*Hlx1*), for sequences matching the PRDM9^b^ and the PRDM9^wm7^ motifs. That was done with the FIMO program (http://meme.nbcr.net/meme4_6_1/). The table shows the sequences matching these motifs with a *p* value smaller than 10^−3^. The motif located in a 200 bp window centered on *Hlx1* hotspot center is in bold. Intervals covered by the probes (NCBI m37 mouse genome assembly). *G7c*, probes 1–5: Chr17, 35,156,465–35,157,586. *G7c*, probe 6: Chr17, 35,157,547–35,157,829. *G7c*, probes 7–10: Chr17, 35,157,789–35,158,670. *Psmb9*, probes 1–3: Chr17, 34,316,603–34,317,193. *Psmb9*, probe 4: Chr17, 34,317,139–34,317,339. *Psmb9*, probes 5–7: Chr17, 34,317,307–34,317,863. *Hlx1*, PRDM9^wm7^ binding motif: Chr1, 186,440,863–186,440,893.
(DOC)

Table S9 Number of predicted PRDM9^b^ and PRDM9^wm7^ binding sequences with a *p* value lower than 10^−3^ at *G7c*, *Psmb9*, and *Hlx1* hotspots. This table recapitulates the number of motifs (shown in A) with a *p* value<10^−3^ found either on the probe positive for PRDM9^b^ (*G7c*, probe 6) or PRDM9^wm7^ (*Psmb9*, probe 4), or on the intervals covered by probes that fail to show any evidence for PRDM9 binding (*G7c*, probes 1–5 and 7–10, *Psmb9*, probes 1–3 and 5–7; Figures 2 and 3). At *Hlx1*, windows extending 100 bp and 1,000 bp on both sides of the motif that binds PRDM9^wm7^ in vitro (Figure 2C) were analyzed.
(DOC)

Table S10 Statistical analysis of H3K4me3 enrichment at *Psmb9* and *Hlx1* hotspots in testes of 6, 9, 12, and 15 d post-partum (dpp) old mice. The level of H3K4 enrichment at *Psmb9* and *Hlx1* hotspots in testes from 9 dpp, 12 dpp, and 15 dpp was compared to that of 6 dpp old males. Stars indicate significant statistical difference (*p*<0.05) between time points.
(DOC)

Table S11 H3K4me3 enrichment in testes from prepuberal R209 mice at *Psmb9* and *Hlx1* hotspots. The values in Table S11 are the bound fraction for each STS, normalized to the bound fraction for STS Psmb9-1, as described in [2].
(DOC)

Table S12 Primers for engineering the Tg(*wm7*) BAC transgene.
(DOC)

Table S13 Primers for cloning the *Prdm9* cDNA.
(DOC)

Table S14 Oligonucleotides used for preparing southwestern probes.
(DOC)

Table S15 Allele-specific primers used for measuring exchanges at the *G7c* hotspot.
(DOC)

Table S16 RT-PCR primers.
(DOC)

Text S1 Prediction of PRDM9 binding sequences in *G7c*, *Hlx1* and *Psmb9* hotspots.
(DOC)

## Abbreviations

BAC: bacterial artificial chromosome
COs: crossovers
DSB: double-strand break
FISH: fluorescent in situ hybridization
IF: immunofluorescence
NCOs: non-crossovers
SNPs: single nucleotide polymorphisms
