# Supplementary figures and images for: Mouse PRDM9 DNA-Binding Specificity Determines Sites of Histone H3 Lysine 4 Trimethylation for Initiation of Meiotic Recombination
Source: PLoS Biol. 2011 Oct 18;9(10):e1001176. doi: 10.1371/journal.pbio.1001176 (PMC3196474; doi:10.1371/journal.pbio.1001176)

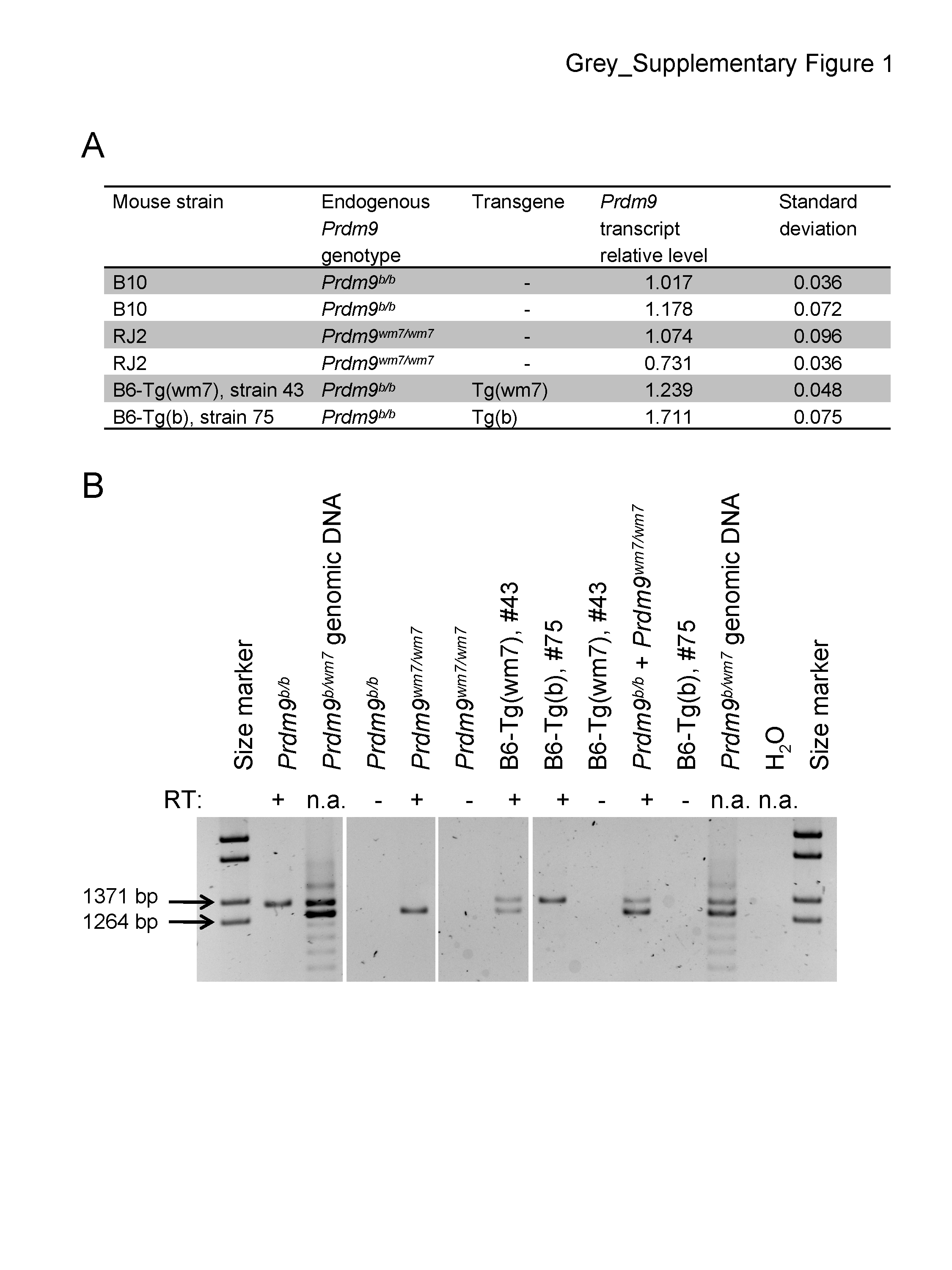

Supplement: Figure S1 — Expression of transgenic Prdm9 copies. (A) The level of Prdm9 transcript in total RNA from elutriated testis cells was measured by RT-qPCR, using Gapdh, Hprt, Actin, and Spo11 as references. The ratio was normalized to 1 for the average of the four samples from strains without a transgene (B10 and RJ2). The two B10 and RJ2 samples are independent preparations from different mice of the same genotype. (B) A 1,371 bp (allele b) or 1,287 bp (allele wm7) fragment of Prdm9 cDNA was amplified from several cDNA samples and run on an agarose gel. Controls without reverse-transcriptase (RT) show no amplification. The Prdm9b/wm7 genomic DNA sample provides a reference for equimolar concentration of both alleles, showing the more efficient amplification of the smaller wm7 allele. The amounts of product of both alleles appear fairly similar in the sample from the transgenic B6-Tg(wm7) strain #43, indicating that there is slightly more RNA from the endogenous Prdm9b locus than from the Prdm9wm7ZF transgene. Size markers of 1,371 and 1,264 bp are indicated by arrows. n.a., not applicable. (TIF) [file pbio.1001176.s001.tif]

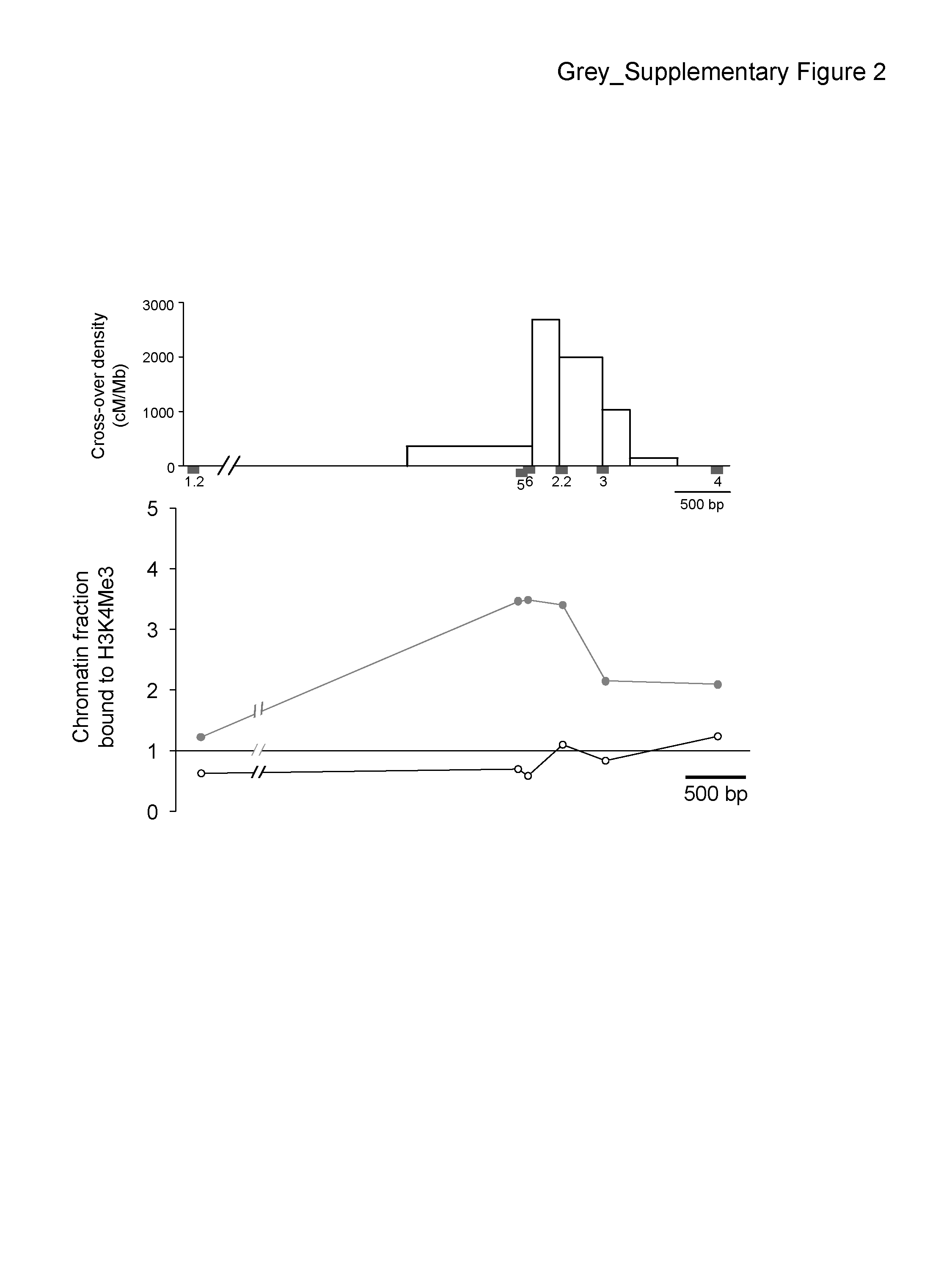

Supplement: Figure S2 — H3K4me3 enrichment at the Hlx1 hotspot is controlled by the PRDM9 zinc finger array. Top panel, distribution of COs and positions of STSs along the Hlx1 hotspot [2]. The chromatin fraction bound to H3K4me3, normalized to Psmb9 STS1 (the 5′ most flanking STS at the Psmb9 hotspot), was determined in elutriated spermatocytes for each STS, as described [2]. Open circles, (B6-Tg(b)×B10.A)F1; gray circles, (B6-Tg(wm7)×B6)F1. (TIF) [file pbio.1001176.s002.tif]

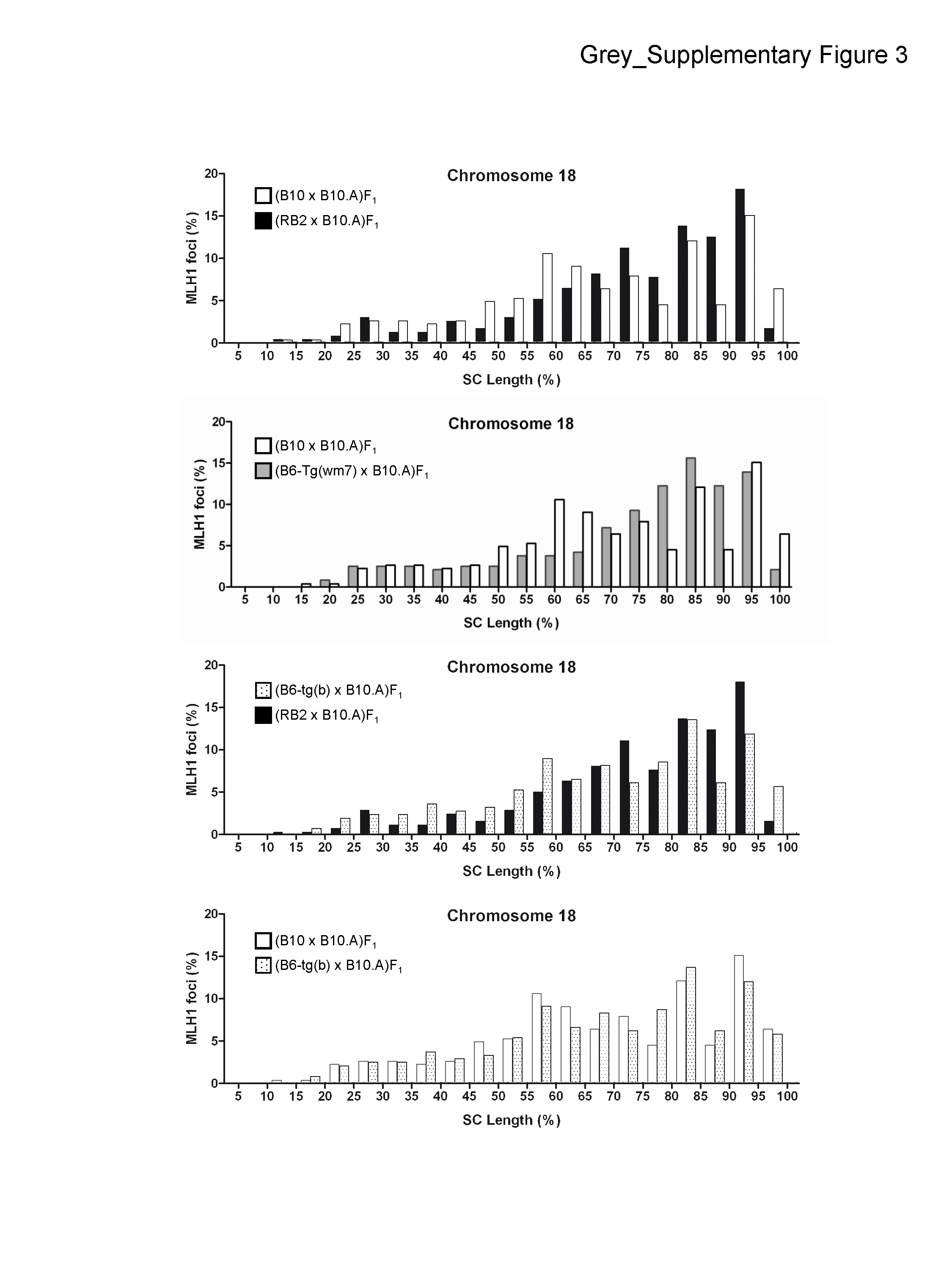

Supplement: Figure S3 — Distribution of MLH1 foci along chromosome 18. The distribution of MLH1 foci along chromosome 18 was determined in pachytene chromosome spreads as described [3]. White, (B10×B10.A)F1, data from [3]; black, (RB2×B10.A)F1, data from [3]; spotted, (B6-Tg(b)×B10.A)F1; gray, (B6-Tg(wm7)×B10.A)F1. (TIF) [file pbio.1001176.s003.tif]

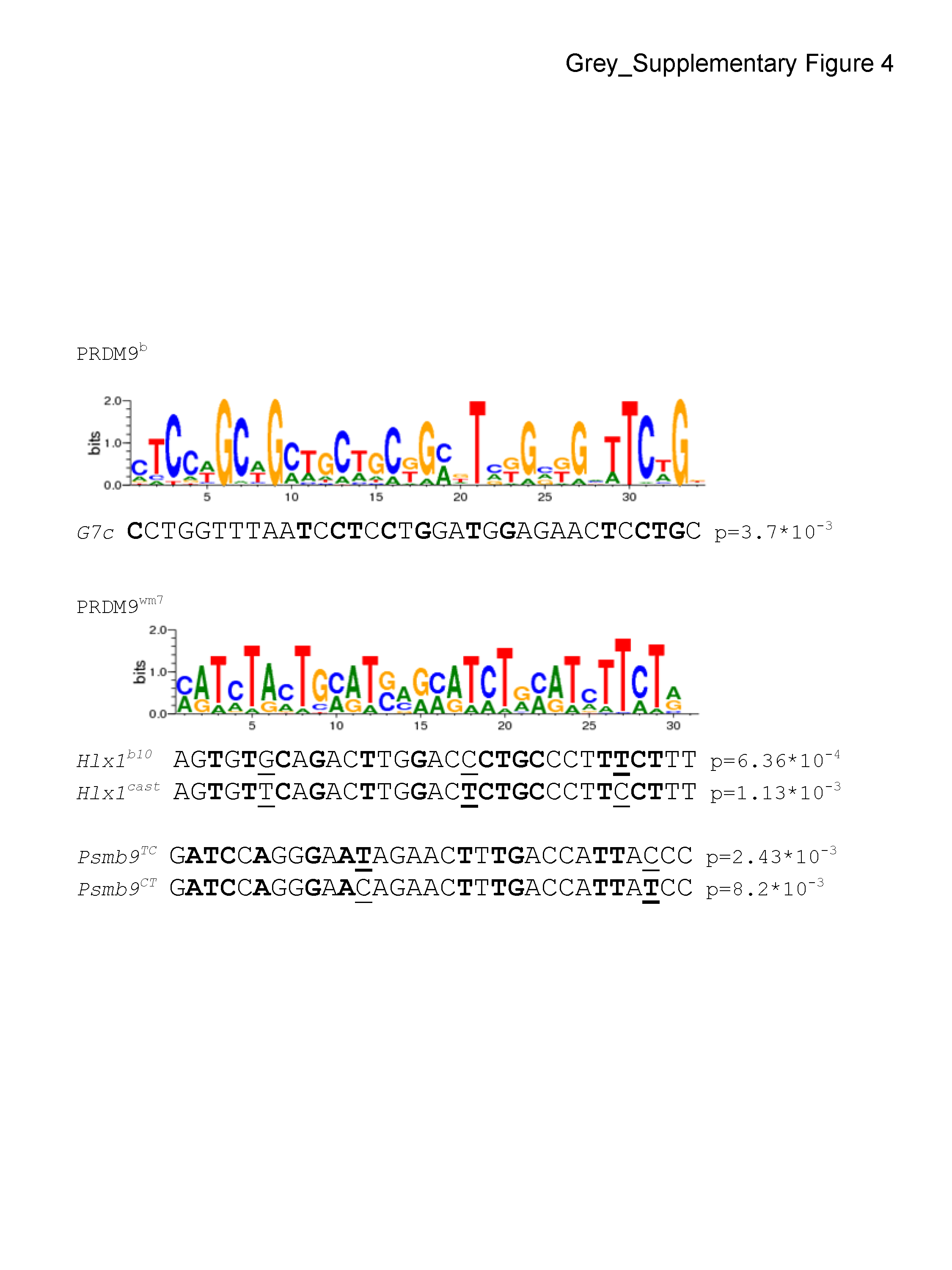

Supplement: Figure S4 — Prediction of DNA sequence motifs recognized preferentially by PRDM9b and PRDM9wm7. The predictions of the DNA binding sequences of PRDM9b and PRDM9wm7 were generated using the program developed by Persikov et al. (http://zf.princeton.edu/) [1]. The logos for the sequences predicted to bind PRDM9b and PRDM9wm7 are shown. Under the PRDM9b logo is the best matching sequence in the interval covered by the G7c probe 6, which binds PRDM9 in vitro (Figure 3). The sequences bound in vitro to PRDM9wm7 at the center of hotspots Psmb9 and Hlx1 are aligned under the PRDM9wm7 logo. The matching residues are in bold, and the polymorphisms affecting PRDM9 binding in vitro and recombination initiation in vivo are underlined (see Figure 2B and C). The p values given by the FIMO program are shown. (TIF) [file pbio.1001176.s004.tif]

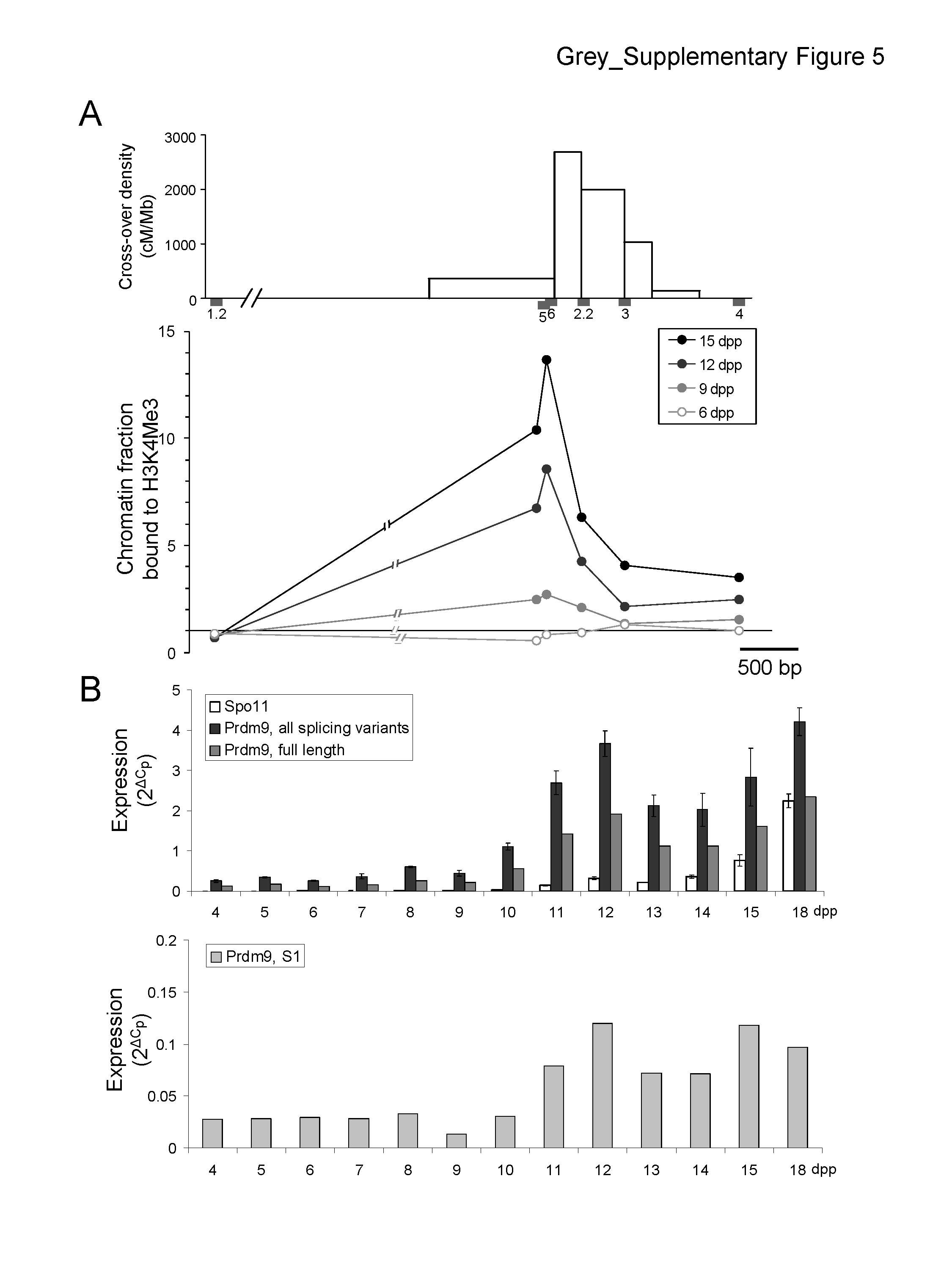

Supplement: Figure S5 — Kinetics of H3K4 trimethylation at Hlx1 and Prdm9 expression in testes of prepuberal mice. (A) Top panel, distribution of COs and positions of STSs along the Hlx1 hotspot [2]. The chromatin fraction bound to H3K4me3, normalized to STS1 (the 5′ most flanking STS), was determined along the Hlx1 hotspot in whole testes from prepuberal R209 mice, as described [2]. (B) Top panel, steady-state levels of Spo11 (white) and Prdm9 (all splicing variants, black; full length, grey; S1 splicing variant, white) expression were determined in whole testes from 4 to 18 dpp mice. The relative changes in expression of the S1 variant are also shown in the lower panel in which a scale with a lower order of magnitude was used. (TIF) [file pbio.1001176.s005.tif]
