# Supplementary material for: Mouse PRDM9 DNA-Binding Specificity Determines Sites of Histone H3 Lysine 4 Trimethylation for Initiation of Meiotic Recombination
Source: PLoS Biol. 2011 Oct 18;9(10):e1001176. doi: 10.1371/journal.pbio.1001176 (PMC3196474; doi:10.1371/journal.pbio.1001176)
Supplement: Table S1 — Measurement of CO and NCO at the Psmb9 hotspot in sperm from (B6-Tg×B10.A) F1 mice. CO A-B and CO B-A indicate exchange products in B10.A to B6 and B6 to B10.A orientation, respectively. NCO A→B indicates non-crossover events having taken place on the B10.A chromosome. Reciprocally, NCO B→A indicates the non-crossover events that took place on the B6 chromosome (see [4]). (DOC) [file pbio.1001176.s006.doc]

**Table S1**

| **Strain** | **Mouse id#** | **DNA pools** | **Amplifiable genomes per pool** | **Frequency of recombination events**  **(number of positive DNA pools)** | | | |
| --- | --- | --- | --- | --- | --- | --- | --- |
|  |  |  |  | **COA-B** | **COB-A** | **NCOA🡪B** | **NCOB🡪A** |
| B6-Tg(wm7) #43 | 139102 | 48 | 227 | 0.46%  (31) | 0.25%  (21) | 0.46%  (31) | 0.36%  (27) |
| B6-Tg(wm7) #43 | 139103 | 48 | 196 | 0.47%  (29) | 0.50%  (30) | 0.42%  (27) | 0.75%  (36) |
| B6-Tg(b) #95 | 32537 | 48 | 270 | 0 | 0 | 0 | 0 |
| B6-Tg(b) #55 | 32386 | 48 | 332 | 0 | 0 | 0 | 0 |
