# Supplementary material for: Mouse PRDM9 DNA-Binding Specificity Determines Sites of Histone H3 Lysine 4 Trimethylation for Initiation of Meiotic Recombination
Source: PLoS Biol. 2011 Oct 18;9(10):e1001176. doi: 10.1371/journal.pbio.1001176 (PMC3196474; doi:10.1371/journal.pbio.1001176)
Supplement: Table S2 — Statistical analysis of H3K4me3 enrichment in elutriated spermatocytes from transgenic mice. Inter-genotype statistical analysis of H3K4me3 enrichment (values shown on Table S3) in elutriated spermatocytes on Psmb9 and Hlx1 hotspots. Stars indicate significant statistical difference (p<0.05) between the genotypes. Data for B6 and R209 were imported from [2]. The level of H3K4 enrichment at Psmb9 and Hlx1 hotspots in purified spermatocytes was compared between transgenic mice and non-transgenic mice of various Prdm9 genotypes. a The difference observed at Hlx1 reflects lower H3K4me3 enrichments in B6-Tg (b)×B10.A as compared to B6 (see values in Table S3). (DOC) [file pbio.1001176.s007.doc]

**Table S2**

|  | p-value 2-sided Mann-Whitney test | |
| --- | --- | --- |
| Hybrids | *Psmb9 locus* | *Hlx1 locus* |
| B6-Tg (wm7) x B6 vs B6-Tg(b) x B10.A | <0.0001* | <0.0001* |
| B6-Tg (wm7) x B6 vs B6 | <0.0001* | 0.0065* |
| B6-Tg (wm7) x B6 vs R209 | 0.3735 | 0.322 |
| B6-Tg (b) x B10.A vs B6 | 0.0703 | 0.003* ^a^ |
| B6-Tg (b) x B10.A vs R209 | <0.0001* | 0.0001* |
