# Supplementary material for: Mouse PRDM9 DNA-Binding Specificity Determines Sites of Histone H3 Lysine 4 Trimethylation for Initiation of Meiotic Recombination
Source: PLoS Biol. 2011 Oct 18;9(10):e1001176. doi: 10.1371/journal.pbio.1001176 (PMC3196474; doi:10.1371/journal.pbio.1001176)
Supplement: Table S3 — H3K4me3 enrichment in elutriated spermatocytes from transgenic mice at hotspots Psmb9 and Hlx1. The values in Table S3 are the bound fraction for each STS, normalized to the bound fraction for STS Psmb9-1, as described in [2]. B6 and R209 data are from [2]. (DOC) [file pbio.1001176.s008.doc]

**Table 3**

| STS | B6 | B6-Tg(b) #75 | R209 | B6-Tg(wm7) #43 |
| --- | --- | --- | --- | --- |
| Psmb9-7 | 1.475  1.169  1.085 | 0.769  0.660  0.732  0.701  1.018 | 2.382  2.676  3.970 | 1.520  3.180  2.873  1.678  1.687  2.127 |
| Psmb9-8 | 1.104  1.238  0.636 | 0.996  1.043  1.069  1.007  1.193 | 3.272  3.789  5.757 | 2.422  4.592  5.776  4.535  3.381  4.010 |
| Psmb9-11 | 1.447  1.001  0.794 | 0.954  0.919  0.724  0.805  1.153 | 2.832  2.956  3.796 | 1.592  3.366  3.636  3.038  2.026  2.719 |
| Psmb9-13 | 1.955  0.586  1.622 | 1.346  0.873  1.046  1.016  0.999 | 1.941  1.713  2.280 | 1.998  2.253  2.553  2.792  1.573  2.047 |
| Hlx1-5 | 1.572 | 0.775  0.652  0.664  0.757  0.584 | 3.983  2.796 | 1.535  4.616  5.491  3.950  2.336  2.853 |
| Hlx1-6 | 1.672  2.540 | 0.615  0.550  0.548  0.567  0.608 | 3.222  3.655  6.641 | 1.870  4.823  5.038  3.571  2.214  3.389 |
| Hlx1-2.2 | 1.304  1.079 | 1.176  0.978  1.127  1.205  0.971 | 3.684  4.559  4.047 | 2.159  4.439  4.596  3.614  2.033  3.549 |
