# Supplementary material for: Mouse PRDM9 DNA-Binding Specificity Determines Sites of Histone H3 Lysine 4 Trimethylation for Initiation of Meiotic Recombination
Source: PLoS Biol. 2011 Oct 18;9(10):e1001176. doi: 10.1371/journal.pbio.1001176 (PMC3196474; doi:10.1371/journal.pbio.1001176)
Supplement: Table S4 — Statistical analysis of the variation between mouse strains in the MLH1 focus distribution on chromosome 18. The distribution of MLH1 foci along chromosome 18 synaptonemal complex (SC) was compared between spermatocytes from mice with different genotypes, using a nonparametric Kolmogorov-Smirnov test and a chi-square test. Stars indicate significant statistical difference (p<0.05) between genotypes. We showed previously for (B10×B10.A) and (RB2×B10.A) F1 hybrids that the distribution of MLH1 foci did not vary significantly between individuals of the same genotype (see Table S7 in [3]). Data for B10×B10.A and RB2×B10.A were imported from [3]. (DOC) [file pbio.1001176.s009.doc]

**Table S4**

| Hybrids | p-value Kolmogorov-Smirnov | p-value  Chi Square |
| --- | --- | --- |
| RB2 x B10.A vs B10 x B10.A | 0.001* | 0.002* |
| B6-Tg (wm7) x B10.A vs B6-Tg(b) x B10.A | 0.018* | 0.111 |
| B6-Tg (wm7) x B10.A vs B10 x B10.A | 0.007* | 0.023* |
| B6-Tg (wm7) x B10.A vs RB2 x B10.A | 0.869 | 0.840 |
| B6-Tg (b) x B10.A vs RB2 x B10.A | 0.006* | <0.001* |
| B6-Tg (b) x B10.A vs B10 x B10.A | 0.665 | 0.801 |
