# Supplementary material for: Mouse PRDM9 DNA-Binding Specificity Determines Sites of Histone H3 Lysine 4 Trimethylation for Initiation of Meiotic Recombination
Source: PLoS Biol. 2011 Oct 18;9(10):e1001176. doi: 10.1371/journal.pbio.1001176 (PMC3196474; doi:10.1371/journal.pbio.1001176)
Supplement: Table S5 — SC lengths, average, and total MLH1 focus number on chromosome 18. Data for B10×B10.A and RB2×B10.A were imported from [3]. (DOC) [file pbio.1001176.s010.doc]

**Table S5**

| Hybrids | Average MLH1 focus number | SC length  (μm) | n (foci) | n (mice) |
| --- | --- | --- | --- | --- |
| B10 x B10.A | 0.99 ± 0.02 | 6.71 ± 0.17 | 231 | 2 |
| RB2x B10.A | 0.99 ± 0.02 | 5.91 ± 0.10 | 265 | 2 |
| B6-Tg (b) x B10.A | 1.01 ± 0.09 | 5.82 ± 0.59 | 237 | 1 |
| B6-Tg (wm7) x B10.A | 1.02 ± 0.13 | 6.16 ± 0.76 | 241 | 1 |
