# Supplementary material for: Mouse PRDM9 DNA-Binding Specificity Determines Sites of Histone H3 Lysine 4 Trimethylation for Initiation of Meiotic Recombination
Source: PLoS Biol. 2011 Oct 18;9(10):e1001176. doi: 10.1371/journal.pbio.1001176 (PMC3196474; doi:10.1371/journal.pbio.1001176)
Supplement: Table S6 — Distributions of MLH1 foci on chromosome 18 in transgenic mice. The number of MLH1 foci per 5% interval of chromosome 18 synaptonemal complex length is shown for B6-Tg(b)xB10.A and B6-Tg(wm7)xB10.A mice. (DOC) [file pbio.1001176.s011.doc]

**Table S6**

|  | Focus number per interval | |  |
| --- | --- | --- | --- |
| Interval on SC (%) | B6-Tg (b) x B10.A | B6-Tg (wm7) x B10.A | |
| 0-5 | 0 | 0 | |
| 5-10 | 0 | 0 | |
| 10-15 | 0 | 0 | |
| 15-20 | 2 | 2 | |
| 20-25 | 6 | 5 | |
| 25-30 | 6 | 6 | |
| 30-35 | 6 | 6 | |
| 35-40 | 5 | 9 | |
| 40-45 | 6 | 7 | |
| 45-50 | 6 | 8 | |
| 50-55 | 9 | 13 | |
| 55-60 | 9 | 22 | |
| 60-65 | 10 | 16 | |
| 65-70 | 17 | 20 | |
| 70-75 | 22 | 15 | |
| 75-80 | 29 | 21 | |
| 80-85 | 37 | 33 | |
| 85-90 | 29 | 15 | |
| 90-95 | 33 | 29 | |
| 95-100 | 5 | 14 | |
| Total focus number | 237 | 241 | |
