# Supplementary material for: Mouse PRDM9 DNA-Binding Specificity Determines Sites of Histone H3 Lysine 4 Trimethylation for Initiation of Meiotic Recombination
Source: PLoS Biol. 2011 Oct 18;9(10):e1001176. doi: 10.1371/journal.pbio.1001176 (PMC3196474; doi:10.1371/journal.pbio.1001176)
Supplement: Table S7 — Exchange frequency at the G7c hotspot depends on the Prdm9 allele. (DOC) [file pbio.1001176.s012.doc]

**Table S7**

| Hybrid | *Prdm9* genotype | *G7c* genotype | Exchange Frequency at *G7c* (%)  (B10 to B10.A orientation)  (95% confidence interval) |
| --- | --- | --- | --- |
| B10 x B10.A | *Prdm9^b/b^* | *G7c^b/a^* | 0.062 ± 0.034 |
| RJ2 x R209 | *Prdm9^wm7/wm7^* | *G7c^b/a^* | <0.0018 |
