# Supplementary material for: Mouse PRDM9 DNA-Binding Specificity Determines Sites of Histone H3 Lysine 4 Trimethylation for Initiation of Meiotic Recombination
Source: PLoS Biol. 2011 Oct 18;9(10):e1001176. doi: 10.1371/journal.pbio.1001176 (PMC3196474; doi:10.1371/journal.pbio.1001176)
Supplement: Table S8 — Predicted PRDM9b and PRDM9wm7 binding sequences with a p value lower than 10−3 at G7c, Psmb9, and Hlx1 hotspots. The scoring matrices resulting from the predictions (see Text S1 and Figure S4) were used for searching the intervals that have been probed by South-Western blot (G7c and Psmb9), or a 2 kb interval centered on the hotspot center (Hlx1), for sequences matching the PRDM9b and the PRDM9wm7 motifs. That was done with the FIMO program (http://meme.nbcr.net/meme4_6_1/). The table shows the sequences matching these motifs with a p value smaller than 10−3. The motif located in a 200 bp window centered on Hlx1 hotspot center is in bold. Intervals covered by the probes (NCBI m37 mouse genome assembly). G7c, probes 1–5: Chr17, 35,156,465–35,157,586. G7c, probe 6: Chr17, 35,157,547–35,157,829. G7c, probes 7–10: Chr17, 35,157,789–35,158,670. Psmb9, probes 1–3: Chr17, 34,316,603–34,317,193. Psmb9, probe 4: Chr17, 34,317,139–34,317,339. Psmb9, probes 5–7: Chr17, 34,317,307–34,317,863. Hlx1, PRDM9wm7 binding motif: Chr1, 186,440,863–186,440,893. (DOC) [file pbio.1001176.s013.doc]

**Table S8**

| **Motif** | **HS** | **Chr** | **Start** | **End** | **p-value** | **Matched Sequence** |
| --- | --- | --- | --- | --- | --- | --- |
| PRDM9^b^ | *G7c* | 17 | 35157231 | 35157264 | 0.000123 | CCTACCATTTTAGAGATAGCAGAAGCAGCTTATT |
| PRDM9^b^ | *G7c* | 17 | 35156760 | 35156793 | 0.000204 | TCAGCCTCCTGCGAAGCCGCCTGGGCGACAAGGC |
| PRDM9^b^ | *G7c* | 17 | 35156848 | 35156881 | 0.000236 | GCATTCGTCCCCAATGCCTCAGTTCCCGCAGGCA |
| PRDM9^b^ | *G7c* | 17 | 35156832 | 35156865 | 0.000351 | CCTCAGTTCCCGCAGGCAGGAGCACCGACCCATC |
| PRDM9^b^ | *G7c* | 17 | 35157520 | 35157553 | 0.000351 | TCAGCAGCAGCCTGGGGAGCCGGCTCAGCAGGTA |
| PRDM9^b^ | *G7c* | 17 | 35157003 | 35157036 | 0.000579 | CCAGGCTCGCCGCATTGTGGAGCAGCGTCAAGGC |
| PRDM9^b^ | *G7c* | 17 | 35158534 | 35158567 | 0.00085 | AGCGCCTCTCCCGAGTCAGGGATTCCACCCGGTT |
| PRDM9^b^ | *G7c* | 17 | 35156767 | 35156800 | 0.000951 | GGAGAAAGCCTTGTCGCCCAGGCGGCTTCGCAGG |
| PRDM9^b^ | *Hlx1* | 1 | 186442638 | 186442671 | 0.00031 | CCTGCCACACAGCAGTCAGTGTTAGCAGATGGTT |
| PRDM9^b^ | *Hlx1* | 1 | 186441162 | 186441195 | 0.000405 | GTATATGTTCTCTAGGCCTTTTAGGAAACATGGC |
| PRDM9^b^ | *Hlx1* | 1 | 186440771 | 186440804 | 0.000504 | ACTGATCTTGCAGAAGACCCAGGTTCAACTCTCA |
| PRDM9^b^ | *Hlx1* | 1 | 186441441 | 186441474 | 0.00059 | CCTGAAGCGGGTGAAGGAGAACGATCAGAAGAAA |
| PRDM9^b^ | *Hlx1* | 1 | 186441005 | 186441038 | 0.000613 | ACGTATATGCTCGGTTTATTAACTTCAGCTTCAC |
| PRDM9^b^ | *Hlx1* | 1 | 186440658 | 186440691 | 0.000933 | TGTGAGGTTATATGTGTATAATCATCTGCATGTT |
| PRDM9^b^ | *Psmb9* | 17 | 34316890 | 34316923 | 0.00015 | TCAGAACCAGCTGTTGCCTGTTTGATAGTTTGTG |
| PRDM9^b^ | *Psmb9* | 17 | 34317825 | 34317858 | 0.000298 | CCACAAGCACCCGGTTTCAAAGGAGCGGGACCAT |
| PRDM9^b^ | *Psmb9* | 17 | 34317773 | 34317806 | 0.00031 | AATTAAACAACAGAGTTAGAAGCAGATCCAGGCA |
| PRDM9^b^ | *Psmb9* | 17 | 34317583 | 34317616 | 0.000421 | CCCGGTGCACCCTGCTCAACTGCAGCCAGGAGGG |
| PRDM9^b^ | *Psmb9* | 17 | 34317480 | 34317513 | 0.000773 | CCTCAAGCCTGTGACATGTCTGAACCAGCACCAC |
| PRDM9^wm7^ | *G7c* | 17 | 35156702 | 35156732 | 0.000362 | CACCATAAGCTGCACCTCCAGGCTGCAGAAG |
| PRDM9^wm7^ | *G7c* | 17 | 35158421 | 35158451 | 0.000543 | CCAGGAGAAAAAAAGGCTCATGTTTAAAATG |
| PRDM9^wm7^ | *G7c* | 17 | 35158222 | 35158252 | 0.000715 | GGATAAGAGGGAAAGGGAGAGGCGGGAGAGG |
| PRDM9^wm7^ | *Hlx1* | 1 | 186440469 | 186440499 | 0.000123 | ACTAACTACATAGAGCTGTAAGCTTCAACAG |
| **PRDM9^wm7^** | ***Hlx1*** | **1** | **186440863** | **186440893** | **0.000636** | **AAAGAAAGGGCAGGGTCCAAGTCTGCACACT** |
| PRDM9^wm7^ | *Hlx1* | 1 | 186441441 | 186441471 | 0.000866 | CCTGAAGCGGGTGAAGGAGAACGATCAGAAG |
| PRDM9^wm7^ | *Psmb9* | 17 | 34316734 | 34316764 | 0.000729 | ATTGCTTTAGTACAAGTATAAACCACTCAGT |
| PRDM9^wm7^ | *Psmb9* | 17 | 34317491 | 34317521 | 0.000934 | TCAGACATGTCACAGGCTTGAGGTGTTAAGG |
