# Supplementary material for: Mouse PRDM9 DNA-Binding Specificity Determines Sites of Histone H3 Lysine 4 Trimethylation for Initiation of Meiotic Recombination
Source: PLoS Biol. 2011 Oct 18;9(10):e1001176. doi: 10.1371/journal.pbio.1001176 (PMC3196474; doi:10.1371/journal.pbio.1001176)
Supplement: Table S9 — Number of predicted PRDM9b and PRDM9wm7 binding sequences with a p value lower than 10−3 at G7c, Psmb9, and Hlx1 hotspots. This table recapitulates the number of motifs (shown in A) with a p value<10−3 found either on the probe positive for PRDM9b (G7c, probe 6) or PRDM9wm7 (Psmb9, probe 4), or on the intervals covered by probes that fail to show any evidence for PRDM9 binding (G7c, probes 1–5 and 7–10, Psmb9, probes 1–3 and 5–7; Figures 2 and 3). At Hlx1, windows extending 100 bp and 1,000 bp on both sides of the motif that binds PRDM9wm7 in vitro (Figure 2C) were analyzed. (DOC) [file pbio.1001176.s014.doc]

**Table S9**

|  | | | Motif | |
| --- | --- | --- | --- | --- |
| HS | Interval(s) | Length (bp) | PRDM9^b^ | PRDM9^wm7^ |
| G7c | Probe 6 | 250 | 0 | 0 |
| G7c | Probes 1-5  + Probes 7-10 | 1920 | 8 | 3 |
| Psmb9 | Probe 4 | 170 | 0 | 0 |
| Psmb9 | Probes 1-3  + Probes 5-7 | 1060 | 5 | 2 |
| Hlx1 | Center ± 100 bp | 200 | 1 | 1 |
| Hlx1 | Center ± 1000 bp | 1800 | 4 | 2 |
