# Supplementary material for: Mouse PRDM9 DNA-Binding Specificity Determines Sites of Histone H3 Lysine 4 Trimethylation for Initiation of Meiotic Recombination
Source: PLoS Biol. 2011 Oct 18;9(10):e1001176. doi: 10.1371/journal.pbio.1001176 (PMC3196474; doi:10.1371/journal.pbio.1001176)
Supplement: Table S10 — Statistical analysis of H3K4me3 enrichment at Psmb9 and Hlx1 hotspots in testes of 6, 9, 12, and 15 d post-partum (dpp) old mice. The level of H3K4 enrichment at Psmb9 and Hlx1 hotspots in testes from 9 dpp, 12 dpp, and 15 dpp was compared to that of 6 dpp old males. Stars indicate significant statistical difference (p<0.05) between time points. (DOC) [file pbio.1001176.s015.doc]

**Table S10**

|  | p-value, 2-sided Mann-Whitney test | |
| --- | --- | --- |
| Comparison days post partum | *Psmb9* locus | *Hlx1* locus |
| 9dpp vs 6dpp | 0.0006* | 0.0001* |
| 12dpp vs 6dpp | <0.0001* | <0.0001* |
| 15dpp vs 6dpp | <0.0001* | <0.0001* |
