# Supplementary material for: Mouse PRDM9 DNA-Binding Specificity Determines Sites of Histone H3 Lysine 4 Trimethylation for Initiation of Meiotic Recombination
Source: PLoS Biol. 2011 Oct 18;9(10):e1001176. doi: 10.1371/journal.pbio.1001176 (PMC3196474; doi:10.1371/journal.pbio.1001176)
Supplement: Table S11 — H3K4me3 enrichment in testes from prepuberal R209 mice at Psmb9 and Hlx1 hotspots. The values in Table S11 are the bound fraction for each STS, normalized to the bound fraction for STS Psmb9-1, as described in [2]. (DOC) [file pbio.1001176.s016.doc]

**Table S11**

| STS | 6 dpp | 9 dpp | 12 dpp | 15 dpp |
| --- | --- | --- | --- | --- |
| Psmb9-7 | 0.661  0.692  0.699  1.285 | 1.934  1.691  1.241  1.630 | 6.952  2.422  8.039  3.706 | 11.287  11.783  6.963  5.877 |
| Psmb9-8 | 0.606  0.535  0.604  1.219 | 2.247  2.501  1.100  1.512 | 12.325  3.634  15.010  5.135 | 20.924  16.974  8.438  6.425 |
| Psmb9-11 | 0.390  0.554  0.733  1.318 | 2.148  2.039  1.068  2.140 | 6.646  2.737  7.854  3.356 | 13.021  9.711  5.075  5.011 |
| Psmb9-13 | 1.141  1.048  1.341  2.363 | 1.294  1.522  1.494  0.920 | 4.631  2.156  6.007  3.143 | 6.164  7.092  3.890  3.690 |
| Hlx1-5 | 0.420  0.413  0.426  0.923 | 3.768  3.151  1.172  1.701 | 8.012  3.381  10.704  4.895 | 14.390  12.708  7.741  6.736 |
| Hlx1-6 | 0.529  0.777  0.598  1.407 | 4.148  3.755  1.364  1.604 | 10.393  4.490  13.542  5.854 | 18.042  18.788  10.628  7.151 |
| Hlx1-2.2 | 0.598  0.909  0.805  1.363 | 2.482  2.480  1.425  2.043 | 4.244  2.757  6.570  3.330 | 8.032  7.136  5.639  4.378 |
