# Supplementary material for: Mouse PRDM9 DNA-Binding Specificity Determines Sites of Histone H3 Lysine 4 Trimethylation for Initiation of Meiotic Recombination
Source: PLoS Biol. 2011 Oct 18;9(10):e1001176. doi: 10.1371/journal.pbio.1001176 (PMC3196474; doi:10.1371/journal.pbio.1001176)
Supplement: Table S12 — Primers for engineering the Tg(wm7) BAC transgene. (DOC) [file pbio.1001176.s017.doc]

**Table S12**

| **Name** | **Sequence** |
| --- | --- |
| MsGALKF | attgaaagacaatgtgggcaatatttcagtgataagtcaaatgtcaatgaCCTGTTGACAATTAATCATCGGCA |
| MsGALKR | attgttgagatgtggttttattgctgttggctttctcattcttttcgaaaTCAGCACTGTCCTGCTCCTT |
| Pr1500U20 | ATATGGAATGGAATCATCGC |
| Pr2848L23 | ATTGTTGAGATGTGGTTTTATTG |
