# Supplementary material for: Mouse PRDM9 DNA-Binding Specificity Determines Sites of Histone H3 Lysine 4 Trimethylation for Initiation of Meiotic Recombination
Source: PLoS Biol. 2011 Oct 18;9(10):e1001176. doi: 10.1371/journal.pbio.1001176 (PMC3196474; doi:10.1371/journal.pbio.1001176)
Supplement: Table S13 — Primers for cloning the Prdm9 cDNA. (DOC) [file pbio.1001176.s018.doc]

**Table S13**

| **Name** | **Sequence** |
| --- | --- |
| 1S91U24 | TTCTAGGCTCAGCCCGACCATAGG |
| Pr2848L23 | ATTGTTGAGATGTGGTTTTATTG |
| mPrdm9gwU | GGGGACAAGTTTGTACAAAAAAGCAGGCTTCAACACCAACAAGCTGGAAGA |
| mPrdm9gwL | GGGGACCACTTTGTACAAGAAAGCTGGGTAGGCTTTCTCATTCTTTTCG |
