# Supplementary material for: Mouse PRDM9 DNA-Binding Specificity Determines Sites of Histone H3 Lysine 4 Trimethylation for Initiation of Meiotic Recombination
Source: PLoS Biol. 2011 Oct 18;9(10):e1001176. doi: 10.1371/journal.pbio.1001176 (PMC3196474; doi:10.1371/journal.pbio.1001176)
Supplement: Table S14 — Oligonucleotides used for preparing southwestern probes. (DOC) [file pbio.1001176.s019.doc]

**Table S14**

| **Name** | **Forward primer** | **Reverse primer** |
| --- | --- | --- |
| Psmb9-Xb1 | gctctagagCTATTTGTTGCCAAGTTGC | gctctagagcTTCCGTCTTTAAATCTCCAT |
| Psmb9-Xb2 | gctctagagcTCGCATTTGGTATTTGAGAT | gctctagagCTGGCAACTTGATGTTGTC |
| Psmb9-Xb3 | gctctagagCATGGTCTTAGATATTTCCACT | gctctAGAGCCCTTGAAGCTGTC |
| Psmb9-Xb4 | gctctagaGTCTGTTGTGCAAGATGC | gctctaGAGACAAAGGGAAACTGAG |
| Psmb9-Xb5 | gctctagagcTTTATTTTTCTCACCCTCAGTTTCC | gctctagagcAACACCTCAAGCCTGTGACAT |
| Psmb9-Xb6 | gctctagagcGTGTGGTGCTGGTTCAGA | gctctagagcAGGATTCAGAAAGCAGGAACT |
| Psmb9-Xb7 | gctctagaGCTGGGAGTTCTGACTCTGCT | gctctagagcTCTGTATGGTCCCGCTCCTT |
| G7c-Xb1 | gctctagagcGATGCACACAGAATGTCACTAA | gctctagagCTGGAGGTGCAGCTTATG |
| G7c-Xb2 | gctctagagcGACAGCACATCCCCAAGTTTCT | gctctagagCAGTGAAGTTTCCGCATTCGT |
| G7c-Xb3 | gctctagagcACTGAGGCATTGGGGACGAA | gctctagagCCCTGTAGACCACCGCTACCTG |
| G7c-Xb4 | gctctagagCTTCCACGACCCAGGTAGC | gctctagagCAAAATGAACCCAAAAGGCATA |
| G7c-Xb5 | gctctagagcTTCCTTAGACTTCTGTGCTAAT | gctctagagcACCTCCCCATACACCTT |
| G7c-Xb6 | gctctagagCTGCTGAGGTCAGAGATAAGAT | gctctagagcTGAGGTTATTGACGCATGT |
| G7c-Xb7 | gctctagagcGACTTAGTGTGTGCTTGTGGA | gctctagagCAGGTGGATGAGGAGAATAGAG |
| G7c-Xb8 | gctctagagcTATTGGTATCCACCTCGGGGTA | gctctagagcGGAAGGGCAGGATAAGAGGG |
| G7c-Xb9 | gctctagagCCGCCTCTCCCTTTCCCTCTTA | gctctagagcAGGGGAGGGGTGTGCAACA |
| G7c-Xb10 | gctctagagCCACCATTTTAAACATGAGCCT | gctctagagcGGCCTCCCACTCTGTTAAAG |
| **Name** | **Sequence** | |
| Psmb9TC-U | CTGACCACGTGGATCCAGGGAATAGAACTTTGACCATTACCCACGGGCCATGTTATT | |
| Psmb9TC-L | AAGAAATAACATGGCCCGTGGGTAATGGTCAAAGTTCTATTCCCTGGATCCACGTGG | |
| Psmb9TT-U | CTGACCACGTGGATCCAGGGAATAGAACTTTGACCATTATCCACGGGCCATGTTATT | |
| Psmb9TT-L | AAGAAATAACATGGCCCGTGGATAATGGTCAAAGTTCTATTCCCTGGATCCACGTGG | |
| Psmb9CC-U | CTGACCACGTGGATCCAGGGAACAGAACTTTGACCATTACCCACGGGCCATGTTATT | |
| Psmb9CC-L | AAGAAATAACATGGCCCGTGGGTAATGGTCAAAGTTCTGTTCCCTGGATCCACGTGG | |
| Psmb9CT-U | CTGACCACGTGGATCCAGGGAACAGAACTTTGACCATTATCCACGGGCCATGTTATT | |
| Psmb9CT-L | AAGAAATAACATGGCCCGTGGATAATGGTCAAAGTTCTGTTCCCTGGATCCACGTGG | |
| HlxB-U | TGAATAGTGTGCAGACTTGGACCCTGCCCTTTCTTTAC | |
| HlxB-L | TGCGTAAAGAAAGGGCAGGGTCCAAGTCTGCACACTAT | |
| HlxC-U | TGAATAAGTGTTCAGACTTGGACTCTGCCCTTCCTTTAC | |
| HlxC-L | TGCGTAAAGGAAGGGCAGAGTCCAAGTCTGAACACTTAT | |
