# Supplementary material for: Mouse PRDM9 DNA-Binding Specificity Determines Sites of Histone H3 Lysine 4 Trimethylation for Initiation of Meiotic Recombination
Source: PLoS Biol. 2011 Oct 18;9(10):e1001176. doi: 10.1371/journal.pbio.1001176 (PMC3196474; doi:10.1371/journal.pbio.1001176)
Supplement: Table S15 — Allele-specific primers used for measuring exchanges at the G7c hotspot. (DOC) [file pbio.1001176.s020.doc]

**Table S15**

| **Name** | **Sequence** | **PCR** | **Strain**  **Specificity** | **Annealing**  **Temperature** |
| --- | --- | --- | --- | --- |
| G7C-1UA | ACACATGCGTCAATAACCTCT | 1^st^, 2^nd^ | B10.A, R209 |  |
| G7C-1LB | GCCCTGTGTCCTGCTCTG | 1^st^ | B10, RB2 | 66°C |
| G7C-2LB | AGGATTGAGGGCTAAGGGTC | 2^nd^ | B10, RB2 | 64°C |
| G7C-1UB | ACACATGCGTCAATAACCTCA | 1^st^, 2^nd^ | B10, RB2 |  |
| G7C-1LA | GCCCTGTGTCCTGCTCTA | 1^st^ | B10.A, R209 | 66°C |
| G7C-2LA | AGGATTGAGGGCTGAGGGTT | 2^nd^ | B10.A, R209 | 64°C |
