# Supplementary material for: Mouse PRDM9 DNA-Binding Specificity Determines Sites of Histone H3 Lysine 4 Trimethylation for Initiation of Meiotic Recombination
Source: PLoS Biol. 2011 Oct 18;9(10):e1001176. doi: 10.1371/journal.pbio.1001176 (PMC3196474; doi:10.1371/journal.pbio.1001176)
Supplement: Table S16 — RT-PCR primers. (DOC) [file pbio.1001176.s021.doc]

**Table S16**

| **Name** | **Sequence** | **cDNA dilution** | | **Annealing Temperature** | |
| --- | --- | --- | --- | --- | --- |
|  |  | **kinetics** | **transgenes** | |  |
| Pr1075U21 | ATCTGATCTACCAGTCGGTCT | 1/10 | 1/10 | | 55°C |
| Pr1232L19 | GGGCACAGTTCACATACCT |
| Pr1500U20 | ATATGGAATGGAATCATCGC | 1/10 | 1/10 | | 55°C |
| Pr1675L18 | GTGCTGGGAAAGGTTGTT |  |
| S1-1114U20 | CAACTGGATGAGGTATGTGA | 1/10 | - | | 55°C |
| S1-1323L22 | TTTAAGGAATCATAATAGTGCC |
| S2-1306U20 | CACAGCAGGAAGAGATTTAT | 1/10 | - | | 55°C |
| S2-1450L16 | GAGCACCCGACTGTTC |
| qSpo545U19 | TGAGATACATGGAGGAAGA | 1/10 | 1/10 | | 52°C |
| qSpo698L18 | TGCAGAAGTTGTCGTCCA |
| mGAPDH-F | TGGCAAAGTGGAGATTGTTGCC | 1/1000 | 1/1000 | | 60°C |
| mGAPDH-R | AAGATGGTGATGGGCTTCCCG |
| mHprt-F | AGTCCCAGCGTCGTGATTAGC | 1/1000 | 1/100 | | 62°C |
| mHprt-R | CCAAATCCTCGGCATAATG |
| Act-F | GCCGGCTTACACTGCGCTTCTT | 1/1000 | 1/1000 | | 59°C |
| Act-R | TTCTGGCCCATGCCCACCAT |
