# Supplementary material for: Mouse PRDM9 DNA-Binding Specificity Determines Sites of Histone H3 Lysine 4 Trimethylation for Initiation of Meiotic Recombination
Source: PLoS Biol. 2011 Oct 18;9(10):e1001176. doi: 10.1371/journal.pbio.1001176 (PMC3196474; doi:10.1371/journal.pbio.1001176)
Supplement: Text S1 — Prediction of PRDM9 binding sequences in G7c, Hlx1 and Psmb9 hotspots. (DOC) [file pbio.1001176.s022.doc]

**Supplementary text**

**Prediction of PRDM9 binding sequences in *G7c*, *Hlx1* and *Psmb9* hotspots**.

The program developed by Persikov et al., available online (<http://zf.princeton.edu/>), allows for predicting the DNA binding sequence for C2H2 proteins [1]. We used it for predicting the sequences preferentially bound by PRDM9^b^ and PRDM9^wm7^, using the SVM polynomial model. The logos deduced from the scoring matrices for the sequences predicted to bind PRDM9^b^ and PRDM9^wm7^ are shown on figure S4. These matrices have been used to search genomic interval surrounding the *G7c*, *Psmb9* and *Hlx1* hotspots for sequences matching them, with the FIMO program (<http://meme.nbcr.net/meme4_6_1/>). The intervals covered by the South-western probes have been examined at *Psmb9* (1,261 bp) and *G7c* (2,206 bp) hotspots, (Figures 2A and 3). At *Hlx1*, a 2 kb window centered on the PRDM9^wm7^-binding motif localized at the center of the hotspot (Figure 2C) has been analyzed. All sequences matching the prediction with a p-value lower than 10^-3^ are listed on Table S8. On these intervals representing 5.5 kb overall, 18 and 8 sequences were found matching the PRDM9^b^ and PRDM9^wm7^ motifs, respectively (see Table S9). Of them, only one can bind PRDM9 in vitro in our South-western assay (PRDM9^wm7^ motif at the *Hlx1* hotspot, starting at position 186,440,863 on chromosome 1, which is the second best scoring match on the 2 kb *Hlx1* interval). Therefore, these *in silico* predictions do not allow predicting PRDM9 *in vitro* binding sites. The *in vitro* detected PRDM9^wm7^ binding sites at the center of *Psmb9* (p=2.43x10^-3^) and *Hlx1* (p=6.36x10^-4^), shown on Figure S4, are the best scoring sequences on the 200 bp interval covering the center of these hotspots.

**References from supplemental information**

1. Persikov AV, Osada R, Singh M (2009) Predicting DNA recognition by Cys2His2 zinc finger proteins. Bioinformatics 25: 22-29.

2. Buard J, Barthes P, Grey C, de Massy B (2009) Distinct histone modifications define initiation and repair of meiotic recombination in the mouse. Embo J 28: 2616-2624.

3. Grey C, Baudat F, de Massy B (2009) Genome-Wide Control of the Distribution of Meiotic Recombination. PLoS Biol 7: e35.

4. Baudat F, de Massy B (2007) Cis- and Trans-Acting Elements Regulate the Mouse Psmb9 Meiotic Recombination Hotspot. PLoS Genet 3: e100.
